# The Consequences of Reconfiguring the Ambisense S Genome Segment of Rift Valley Fever Virus on Viral Replication in Mammalian and Mosquito Cells and for Genome Packaging

**DOI:** 10.1371/journal.ppat.1003922

**Published:** 2014-02-13

**Authors:** Benjamin Brennan, Stephen R. Welch, Richard M. Elliott

**Affiliations:** Biomedical Sciences Research Centre, University of St Andrews, North Haugh, St Andrews, Fife, United Kingdom; University of Pennsylvania School of Medicine, United States of America

## Abstract

Rift Valley fever virus (RVFV, family *Bunyaviridae*) is a mosquito-borne pathogen of both livestock and humans, found primarily in Sub-Saharan Africa and the Arabian Peninsula. The viral genome comprises two negative-sense (L and M segments) and one ambisense (S segment) RNAs that encode seven proteins. The S segment encodes the nucleocapsid (N) protein in the negative-sense and a nonstructural (NSs) protein in the positive-sense, though NSs cannot be translated directly from the S segment but rather from a specific subgenomic mRNA. Using reverse genetics we generated a virus, designated rMP12:S-Swap, in which the N protein is expressed from the NSs locus and NSs from the N locus within the genomic S RNA. In cells infected with rMP12:S-Swap NSs is expressed at higher levels with respect to N than in cells infected with the parental rMP12 virus. Despite NSs being the main interferon antagonist and determinant of virulence, growth of rMP12:S-Swap was attenuated in mammalian cells and gave a small plaque phenotype. The increased abundance of the NSs protein did not lead to faster inhibition of host cell protein synthesis or host cell transcription in infected mammalian cells. In cultured mosquito cells, however, infection with rMP12:S-Swap resulted in cell death rather than establishment of persistence as seen with rMP12. Finally, altering the composition of the S segment led to a differential packaging ratio of genomic to antigenomic RNA into rMP12:S-Swap virions. Our results highlight the plasticity of the RVFV genome and provide a useful experimental tool to investigate further the packaging mechanism of the segmented genome.

## Author Summary

Rift Valley fever virus (RVFV) is a mosquito-borne bunyavirus found primarily in sub-Saharan Africa that can infect both domestic animals and humans. RVFV has a tripartite RNA genome that encodes seven proteins. The smallest (S) segment has an unusual ambisense coding strategy whereby two genes (for the nucleocapsid N and nonstructural NSs proteins) are encoded in opposite orientations on the genomic RNA, and are translated from specific subgenomic mRNAs. N is the major structural protein of the virus while NSs is the major virulence factor. To investigate the biological significance of this coding arrangement, we used reverse genetics to create a recombinant virus in which the N and NSs coding sequences were swapped on the S segment. The recombinant virus grew less well in tissue culture cells compared to the parental virus, and rather than maintain persistence in insect cells, infection resulted in their death. In addition, packaging of the modified S genome segment into new virus particles was altered. We also showed that a foreign protein could be expressed to high levels when cloned in place of the NSs gene in the recombinant virus. These studies have implications for vaccine development and vector control strategies.

## Introduction

The *Bunyaviridae* family is composed of five genera: *Orthobunyavirus*, *Hantavirus, Nairovirus*, *Phlebovirus* and *Tospovirus* [1]. Rift Valley fever virus (RVFV) is a member of the *Phlebovirus* genus and is a mosquito-borne pathogen of both livestock and humans that is found primarily in Sub-Saharan Africa and the Arabian Peninsula. In ruminants, RVFV disease is characterised by foetal deformities, abortion and high rates of mortality among young animals that can approach 100% [2]. In humans infection usually results in a self-limiting febrile illness, though on occasion it can develop into retinitis, encephalitis and haemorrhagic disease with an overall 1% case fatality rate [3].

As with the other viruses of the *Phlebovirus* genus, RVFV contains a tripartite RNA genome comprising two negative-sense and one ambisense segments. The large (L) segment encodes the viral RNA-dependent RNA polymerase. The medium (M) segment codes for four proteins in a single open reading frame (ORF): two nonstructural proteins designated NSm1 and NSm2, and the virion envelope glycoproteins Gn and Gc, whose synthesis is dictated by which of five methionine codons are used to initiate translation [4], [5]. The small (S) segment (approx. 1.7 kb) encodes the nucleocapsid protein (N) and a nonstructural protein (NSs) in an ambisense manner. The N protein is translated from a subgenomic mRNA transcribed from the genomic RNA, while NSs is translated from a subgenomic mRNA transcribed from the antigenomic (replicative-intermediate) RNA [6], [7].

The multifunctional NSs protein plays an important role in the pathogenesis of RVFV and acts to overcome the host innate immune response. NSs disrupts host cell metabolism at the transcriptional level by sequestering the p44 subunit and degrading the p62 component of the basal transcription factor TFIIH, while other subunits of the TFIIH core are reduced in infected cells. As a consequence, TFIIH cannot assemble and its concentration drops rapidly within the cell, leading to a drastically reduced transcriptional activity [8], [9]. NSs has also been shown to degrade the double-stranded RNA-dependent protein kinase (PKR) thereby preventing PKR-mediated phosphorylation of the translation initiation factor eIF2a and allowing the continual translation of viral proteins [10], [11]. More recently, the degradation of PKR has been demonstrated to be independent of the NSs-mediated degradation of p62 [12]. To further antagonise host defence mechanisms, NSs also specifically represses transcriptional activation of the interferon (IFN)-β promoter early in infection through its interaction with Sin3A-associated protein 30 (SAP30) mediated by the transcription factor YY1 [13]. This interaction maintains the repressor complex of YY1, SAP30 and other Sin3A-associated factors on the IFN-β promoter. In addition, using a minigenome system, NSs was shown to inhibit the viral polymerase [14] and thus could play a role in regulating viral RNA synthesis. However, despite these multitude of activities, NSs is not essential for replication in either cultured cells or in animals, though viruses lacking NSs are attenuated to various degrees in these systems [15]–[19].

The ambisense S RNA coding strategy adopted by phleboviruses was originally suggested to provide temporal control over gene expression [7], [20]–[23] in that NSs would be translated relatively late in the infectious cycle after replication had commenced with the synthesis of antigenome RNA. However, given that the major function of NSs is as an interferon antagonist, it might be expected that this protein would be required early in infection. Evidence was presented that for Uukuniemi phlebovirus (UUKV) some copies of the S antigenome are packaged into progeny virions [20], and subsequently it was shown that all three antigenome RNAs were packaged into RVFV particles [24]. Furthermore, it was demonstrated that NSs could be translated from mRNA transcribed from infecting antigenome RNA [24]. It was also reported that the ratio of antigenomes to genomes packaged into virions varied from 1∶5 to 1∶100, depending on the cells in which the virus was grown. However, this does not seem an efficient strategy to express NSs early in infection, as not all infectious virions would package the S antigenome.

To investigate the ambisense expression strategy of the RVFV S segment in more detail we asked what the consequences would be of swapping the N and NSs coding sequences on viral replication. To this end we created a recombinant virus by reverse genetics, based on the MP12 attenuated strain of RVFV, in which the N ORF was inserted into the NSs locus and the NSs ORF into the N locus. The virus, called rMP12:S-Swap, thus has an ambisense S segment with N and NSs genes in the opposite orientation to parental MP12 virus. rMP12:S-Swap was attenuated in mammalian cell cultures and had a small plaque phenotype. We show that swapping ORFs on the S genomic RNA led to an increased expression of NSs mRNA and protein in rMP12:S-Swap-infected cells that had implications for the virus' ability to persistently infect mosquito cell lines. Interestingly, in mammalian cells the over-expression of NSs did not lead to an increased inhibition of host cell protein synthesis or degradation of PKR. Rearrangement of the S segment derived ORFs also caused a differential packaging of genomic or antigenomic S RNA species into progeny virions. We discuss the implications of these findings for determining RVFV genome packaging and for understanding the biological role of the RVFV coding strategy in the mammalian host.

## Results

### Generation of recombinant viruses

**Figure 1:**
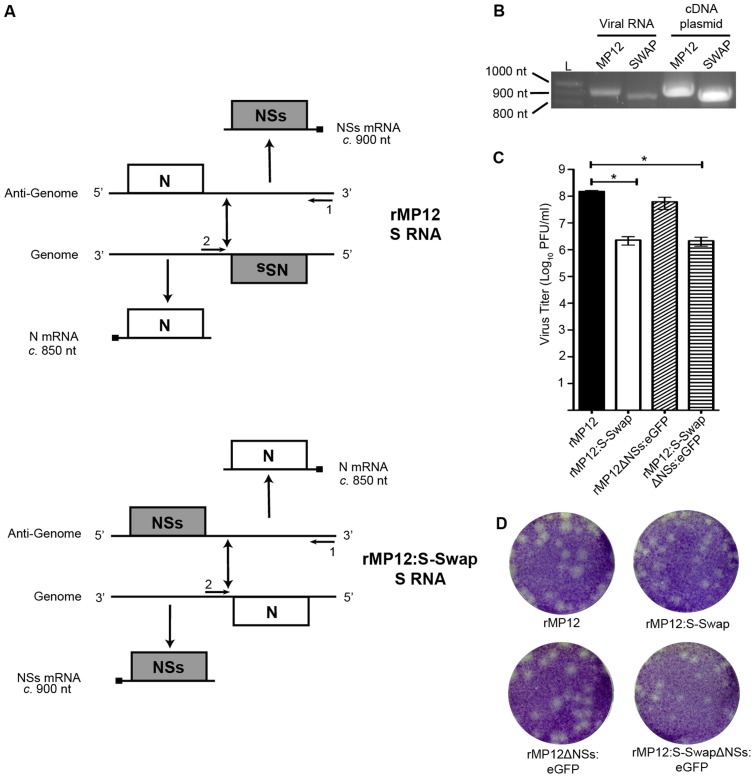
Creation of rMP12:S-Swap. **A.** Schematic of the transcription and replication products of the S segments of rMP12 and rMP12:S-Swap. The sites at which oligonucleotides 1 and 2 anneal are indicated. **B.** Agarose gel showing RT-PCR products to confirm structure of S segment. BHK-21 cells were infected with rMP12 (MP12) or rMP12:S-Swap (SWAP) viruses at an MOI of 1. Total cellular RNA was extracted at 48 h p.i., and S-segment RT-PCR was performed. As a control, PCR on the appropriate cDNA-containing plasmids was performed with the same primers. **C.** Titres of recombinant virus stocks from multiple independent preparations were determined by plaque assay in BHK-21 cells. The mean titre and standard error of n = 4 preparations of each recombinant virus stock are shown (* p>0.05) **D.** Comparison of plaque sizes of rMP12, rMP12:S-Swap, rMP12ΔNSs:eGFP or rMP12:S-SwapΔNSs:eGFP on BHK-21 cells. Cell monolayers were fixed at 96 h p.i. with 4% paraformaldehyde and stained with Giemsa solution.

The ambisense S segment of RVFV comprises 3′ and 5′ terminal untranslated regions, with the coding regions for the N and NSs proteins separated by an untranslated intergenic region that contains the signals for mRNA transcription-termination [25]. The S segment genomic RNA is defined as that in which the N ORF is in the negative-sense and the NSs ORF is in the positive-sense; hence the N mRNA is transcribed from the genomic RNA and the NSs mRNA is transcribed from the antigenomic RNA (Figure 1A). In order to transpose the N and NSs coding sequences an S segment cDNA was synthesised where the N ORF is in the positive-sense at the NSs locus, and the NSs ORF is in the negative-sense at the N locus, but all of the untranslated sequences were untouched (Figure 1A). The cDNA was cloned into pTVT7 [26] to create the plasmid pTVT7-GS:S-Swap. This plasmid was used together with transcription plasmids pTVT7-GL and pTVT7-GM and support plasmids pTM1-N and pTM1-L in the reverse genetics protocol [27]. A recombinant virus was obtained and designated rMP12S:Swap. As a control, a recombinant form of the parental MP12 virus, called rMP12, was recovered at the same time.

An RT-PCR approach was used to confirm the structure of the presumptive swapped S RNA segment within rMP12:S-Swap (Figure 1B). BHK-21 cells were infected with rMP12 or rMP12:S-Swap at a multiplicity of infection (MOI) of 1 and total cellular RNA was extracted at 48 h p.i. The RNA was reverse-transcribed using a segment specific oligonucleotide targeted towards the 5′ end of the S segment (Oligo 1; Figure 1A), and PCR reactions were designed with oligonucleotides to anneal to the 5′ end of the S segment (Oligo 1) or within the S RNA intergenic region (IGR) (Oligo 2). When using Oligos 1 and 2 and rMP12 derived cDNA as a template a product of 918 nt was detected corresponding to the size of the 5′UTR, NSs ORF and IGR. In contrast when rMP12S:Swap cDNA was used a product of 858 nt was detected corresponding to the size of the 5′UTR, N ORF and IGR. The cDNA-containing plasmids pTVT7-GS (parental MP12) and pTVT7-GS:S-Swap (Swap) were used as controls for the RT-PCR product sizes (Figure 1B). Sequence analysis of RT-PCR products confirmed the expected sequence of the reconfigured segment and that no mutations had been introduced during the cloning or virus rescue processes.

We also generated transcription plasmids in which the NSs ORF was replaced with that of enhanced green fluorescent protein (eGFP) in both pTVT7-GS and pTVT7-GS:S-Swap, which were designated pTVT7-GSΔNSs:eGFP and pTVT7-GS:S-SwapΔNSs:eGFP. These constructs were then used in the reverse genetics protocol to generate NSs-deleted forms of the viruses that expressed eGFP, called rMP12ΔNSs:eGFP and rMP12:S-SwapΔNSs:eGFP. Multiple stocks of all recombinant viruses were prepared in BHK-21 cells and had mean titres of 2.28×10^6^ PFU/ml (rMP12S:Swap), 2.15×10^6^ PFU/ml (rMP12:S-SwapΔNSs:eGFP), 1.51×10^8^ PFU/ml (rMP12) and 6.16×10^7^ PFU/ml (rMP12ΔNSs:eGFP). Titres of rMP12:S-Swap and rMP12:S-SwapΔNSs:eGFP were found to be statistically lower (Student's t-test; p>0.05) than that of the parental rMP12 virus (Figure 1C).

The plaque phenotypes of the recombinant viruses produced after 96 h growth in BHK-21 cells are shown in Figure 1D; the plaques produced by rMP12:S-Swap and rMP12:S-SwapΔNSs:eGFP were smaller than those produced by the parental MP12-derived viruses.

### Growth properties of recombinant viruses

**Figure 2:**
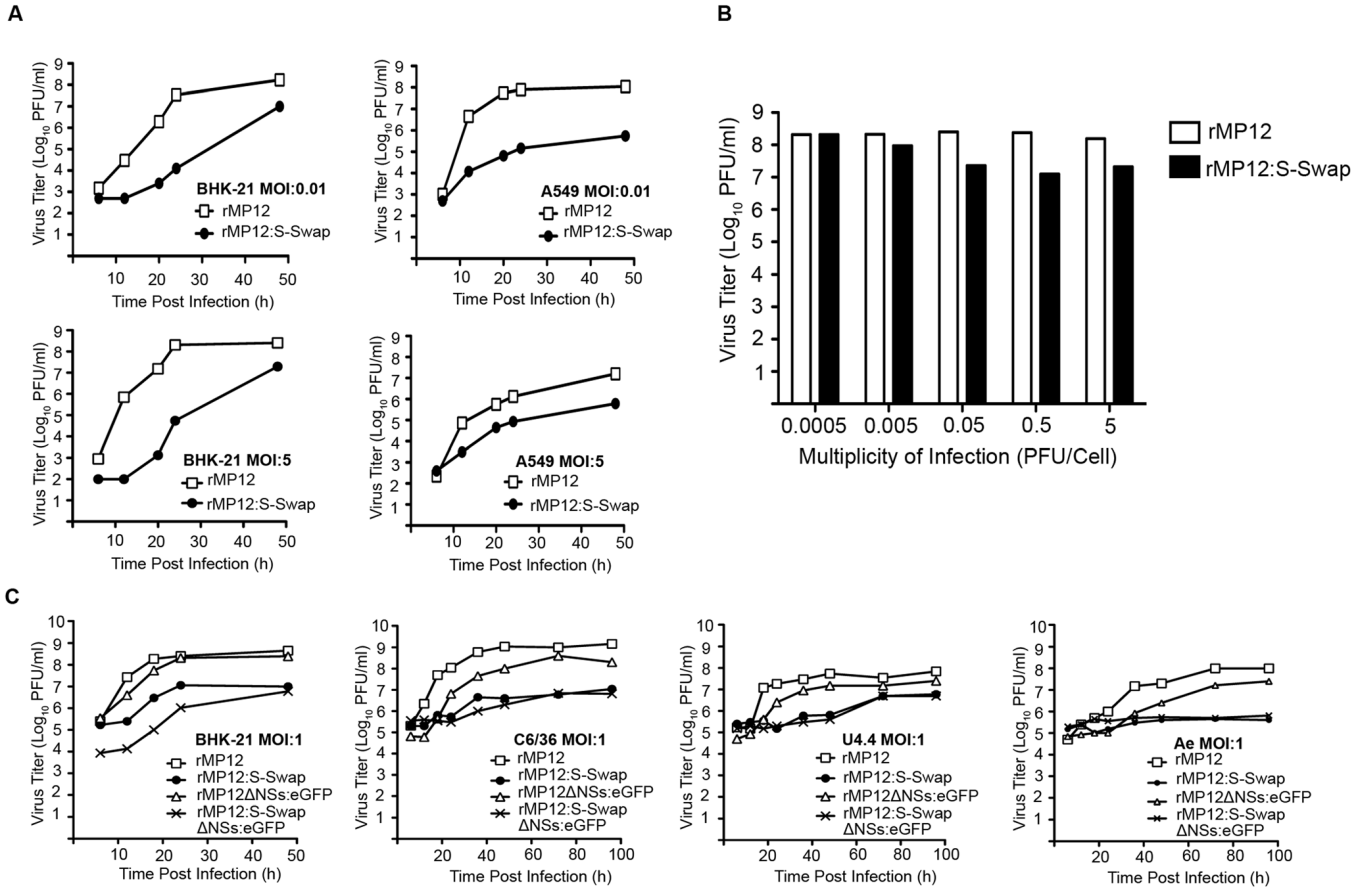
Growth properties of recombinant viruses. **A.** Viral growth curves in BHK-21 and A549 cells infected with rMP12 or rMP12:S-Swap (MOI of 0.01 or 5 as indicated). **B.** The effect of multiplicity of infection on viral yield in BHK-21 cells. Cells were infected with rMP12 or rMP12:S-Swap at multiplicities from 0.0005 to 5 PFU/cell. Viral supernatants were harvested at 72 h p.i. and titrated by plaque assay. Graphs are presented for one representative experiment. **C.** Viral growth curves in mosquito cells. *A. albopictus* C6/36 and U4.4, and *A. aegypti* Ae, cells were infected with rMP12, rMP12:S-Swap, rMP12ΔNSs:eGFP or rMP12:S-SwapΔNSs:eGFP at MOI of 1; BHK-21 cells were similarly infected as a control.

The growth properties of rMP12:S-Swap were compared to those of the parental virus in BHK-21 and A549 cells at high (5 PFU/cell) and low (0.01 PFU/cell) MOI (Figure 2A). At different times post infection the tissue culture supernatants were harvested and virus titres determined by plaque assay in BHK-21 cells. In both cell lines and at both multiplicities of infection the replication of rMP12S:S-Swap was slower than that of rMP12, and peak titres at 48 h p.i. were 10- to 1000-fold lower (Figure 2A). The viruses grew to higher titres in BHK-21 cells (which are interferon incompetent) compared to A549 cells (which are interferon competent) presumably because they did not have to overcome host innate immune defences. It was noted in previous work in our laboratory that for some attenuated viruses, the higher the initial infecting MOI, the lower the resulting titre recorded in certain cell lines [19], [28]. This was thought to be caused by an auto-interference effect associated with the production of defective virus particles [19]. We therefore tested the effect of MOI on virus yield from rMP12- or rMP12:S-Swap-infected BHK-21 cells infected at MOI ranging from 0.0005 to 5 PFU/cell (Figure 2B). At 48 h p.i. tissue culture supernatants were harvested and titrated by plaque assay on BHK-21 cells. As seen previously there was little effect on virus yield of rMP12 infected at the different multiplicities, with yields ranging from 1.55 to 2.55×10^8^ PFU/ml. For rMP12:S-Swap the highest yield obtained (2.1×10^8^ PFU/ml) was from cells infected at the lowest MOI, and at higher multiplicities of infection a decrease in yield by up to 10-fold was observed (Figure 2B).

The growth of recombinant viruses in which the NSs ORF had been replaced with that of eGFP were also examined in mosquito cells (*Aedes albopictus* C6/36 and U4.4 cells, and *Aedes aegypti* Ae cells) at a MOI of 1 (Figure 2C). This was the highest MOI that could be used because mosquito cells are small and thus there are more cells per given area of a tissue culture plate; for comparison BHK-21 cells infected at the same MOI were included. In all cell lines, viruses that contained a swapped S-RNA segment (rMP12:S-Swap or rMP12:S-SwapΔNSs:eGFP) produced 10- to 100-fold less virus over a 48 h or 96 h period. It should be noted that initial titres of virus released from mosquito cells (e.g. at 6 h p.i.) were relatively high in these experiments because it is not possible to completely remove residual inoculum by extensive rinsing as the cells do not adhere firmly to the culture vessels, as noted previously [29].

### Protein synthesis in infected cells

**Figure 3:**
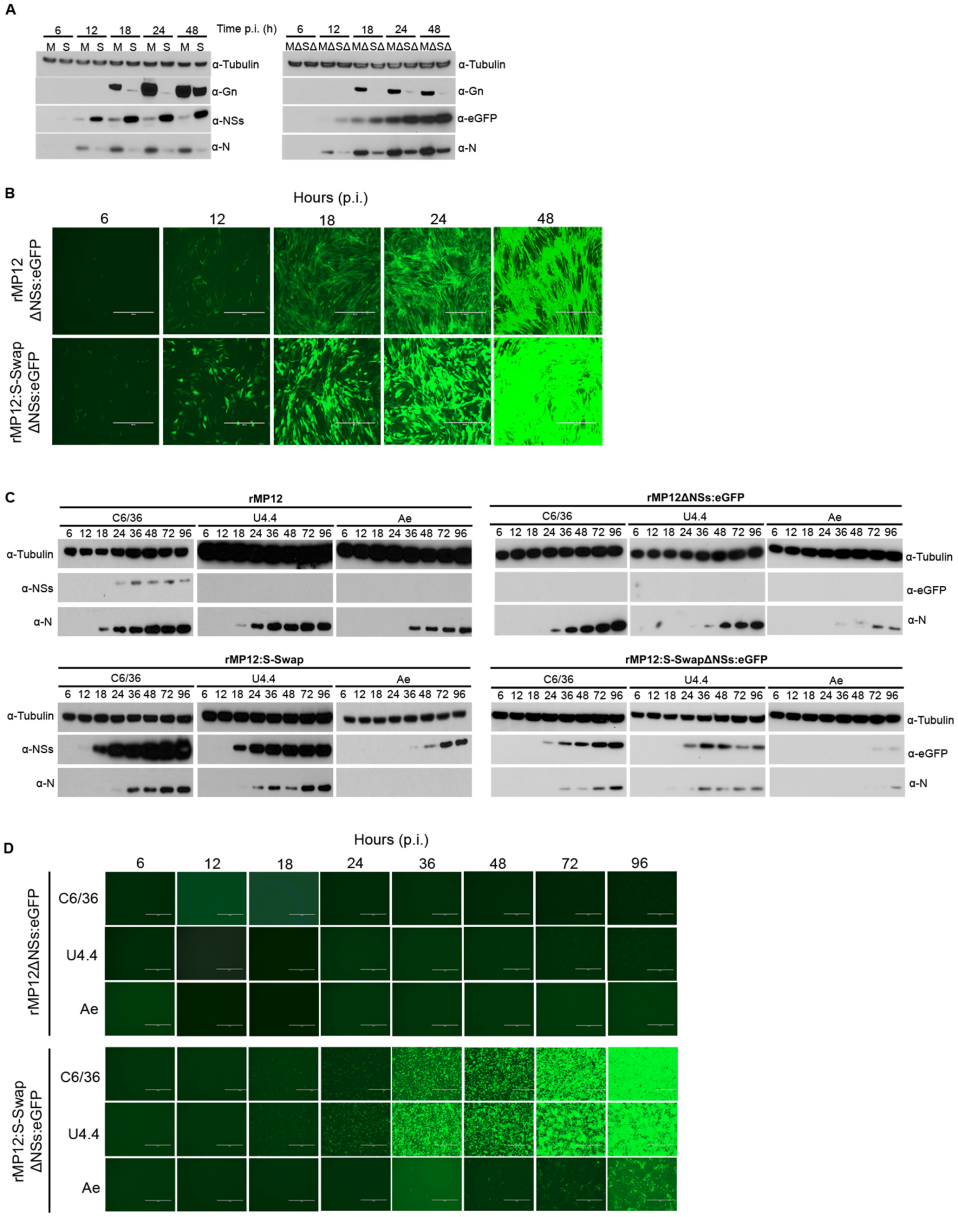
Protein expression by recombinant viruses. **A.** Western blot analysis of proteins synthesised in BHK-21 cells infected with rMP12 (M), rMP12:S-Swap (S), rMP12ΔNSs:eGFP (MΔ) or rMP12:S-SwapΔNSs:eGFP (SΔ) at MOI of 1. Cell lysates, prepared at the indicated h p.i., were fractionated by SDS-PAGE, and after transfer, membranes were reacted with rabbit antibodies specific for N, NSs, Gn or eGFP as indicated. Anti-tubulin antibodies were used as a loading control. **B.** eGFP fluorescence in BHK-21 infected with rMP12ΔNSs:eGFP or rMP12:S-SwapΔNSs:eGFP as above. **C.** Western blot analysis of infected mosquito cells. *A.albopictus* C6/36, U4.4 or *A. aegypti* Ae cells were infected with recombinant viruses (MOI of 1) and lysates prepared at different times post infection. Fractionated proteins were probed with the indicated antibodies. **D.** eGFP fluorescence in mosquito cells infected with rMP12ΔNSs:eGFP or rMP12:S-SwapΔNSs:eGFP as above. Note that eGFP fluorescence in parts B and D was recorded first and then the same cells were harvested for the western blotting.

BHK-21 cells were infected with the recombinant viruses at a MOI of 0.01 and cell monolayers harvested at the time points indicated. Western blotting of cell lysates was performed to monitor the expression of N, NSs, Gn, and, in the case of the NSs deletion viruses, eGFP (Figure 3A). This low MOI was used as preliminary experiments had shown a temporal difference between N and NSs expression in rMP12-infected cells, which was not seen at higher MOI (unpublished observations). In rMP12-infected cells, N protein was clearly detected at 12 h p.i. with NSs protein faintly detectable at this time point (Figure 3A, left panel). The intensity of N and NSs bands increased over the 48 h time course. Conversely, in rMP12:S-Swap-infected BHK-21 cells the NSs band was very obvious at 12 h p.i. whereas the N protein band was barely detectable (Fig 3A, left panel). The intensity of the NSs band increased over 48 h, and was always stronger than NSs in the corresponding time point from rMP12-infected cells. On the other hand, the amount of N detected in rMP12:S-Swap-infected cells was substantially less than that detected in rMP12-infected cells at the same time points and the amount of N protein produced did not increase dramatically over the 48 h period. There was significantly less Gn glycoprotein synthesised in rMP12:S-Swap-infected cells at early time points, and although by 48 h p.i. Gn was clearly detected, its accumulation was less than in rMP12-infected cells (Figure 3A, left panel).

To assess if the difference in N expression between the two viruses could be an effect of the increased early synthesis of NSs (due to the swapping of the N and NSs ORFs) we compared protein synthesis by the two NSs-deleted viruses, rMP12ΔNSs:eGFP and rMP12:S-SwapΔNSs:eGFP, that contain eGFP in the place of the NSs coding sequence (Figure 3A, right panel). Similar to results with NSs-expressing viruses, accumulation of N and Gn was much lower in rMP12:S-SwapΔNSs:eGFP-infected cells, whereas the expression of eGFP mimicked that of NSs. These results suggest that the reduced expression of N and Gn was not due to the amount or timing of NSs production.

We also visualised eGFP fluorescence directly in cells infected with rMP12ΔNSs:eGFP or rMP12:S-SwapΔNSs:eGFP over 48 h. Images were recorded at the same brightness setting on the microscope. At all-time points, the intensity of eGFP fluorescence observed in rMP12:S-SwapΔNSs:eGFP-infected cells was significantly greater than that in rMP12ΔNSs:eGFP-infected cells (Figure 3B). These data corroborate the western blot results shown above.

Protein synthesis was also examined in the mosquito cell lines. No NSs or eGFP protein was detected by western blotting in any cell line infected with rMP12 or rMP12ΔNSs:eGFP other than NSs in C6/36 cells, whereas N protein was readily detectable and increased over the time course in all cell lines (Figure 3C, upper panel). It was noted that N protein expression was rather lower in infected Ae cells compared to the *A. albopictus* cell lines. No eGFP fluorescence was observed in any of the cell lines (Figure 3D, upper panel). However, when mosquito cells were infected with rMP12:S-Swap NSs protein expression was readily detected in U4.4. and Ae cells, and levels of NSs were dramatically increased in C6/36 cells compared to rMP12 infected cells (Figure 3C, lower panel). eGFP protein was also detected in the three mosquito cell lines infected with rMP12:S-SwapΔNSs:eGFP. N protein could also be detected, at lower levels compared to rMP12 infection but its expression was again particularly weak in infected Ae cells. eGFP fluorescence was also observed by microscopic examination of infected cells though was weaker in Ae cells (Figure 3D, lower panel).

### NSs localisation and filament formation

**Figure 4:**
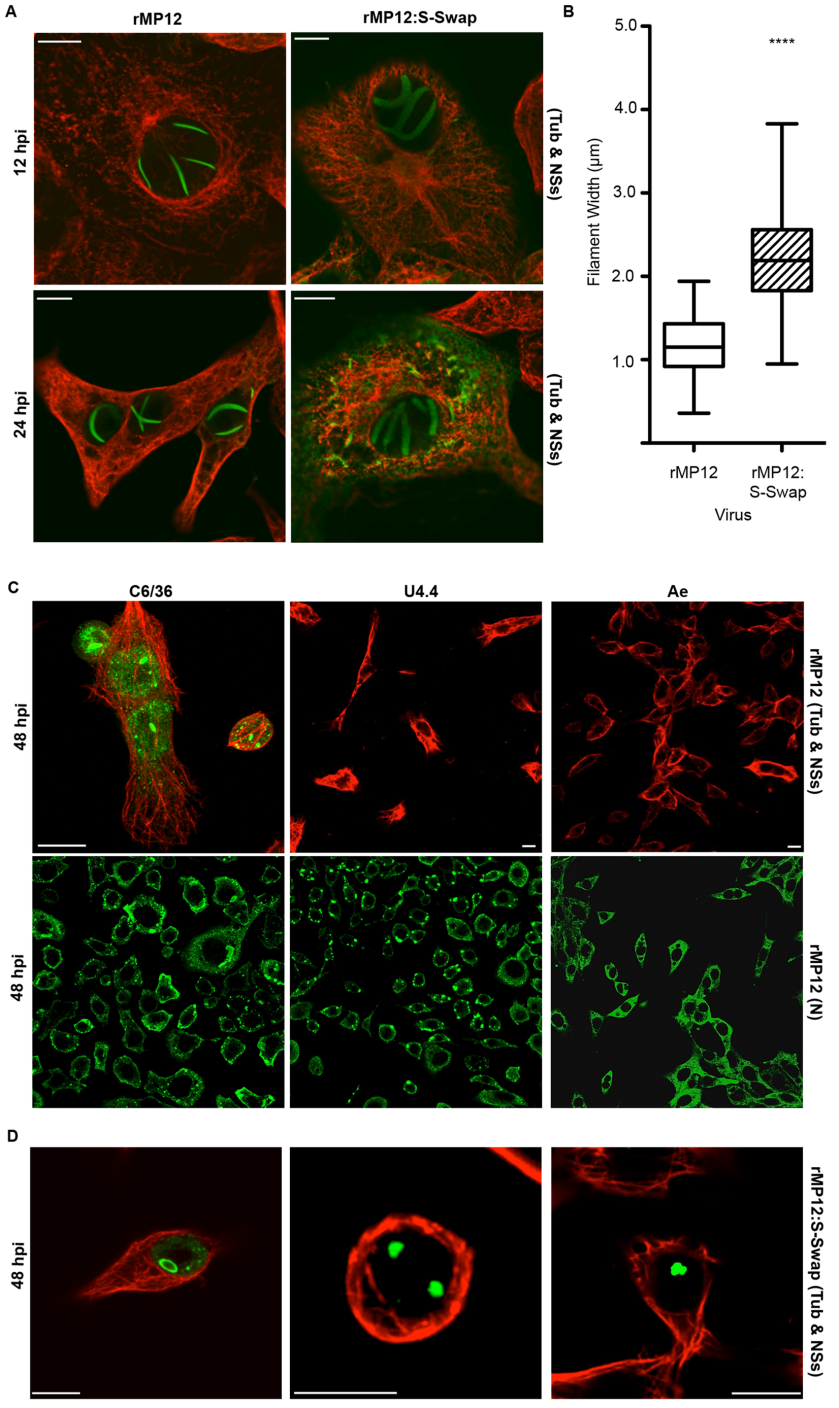
Intracellular localization of NSs in rMP12- or rMP12:S-Swap-infected cells. A. Vero-E6 cells were infected at MOI of 1, and at the time points indicated the cells were fixed with 4% paraformaldehyde, followed by co-staining with anti-NSs (green) and anti-tubulin (red) antibodies. Cells were examined with a Zeiss LSM confocal microscope. **B.** Width of NSs filaments. The widths of NSs filaments (50) in rMP12- or rMP12:S-Swap were measured using the LSM Image Browser Software (Carl Zeiss MicroImaging GmbH) and the results presented as the mean width of the filaments and SEM of the two groups (**** = p<0.0001; see Methods). **C.** Detection of NSs in mosquito cells infected with rMP12. *A.albopictus* C6/36, U4.4 or *A. aegypti* Ae cells were infected with recombinant viruses (MOI of 1), and at 48 h p.i. the cells were fixed with 4% paraformaldehyde, followed by co-staining with anti-NSs (green) and anti-tubulin (red) antibodies (upper panels). Duplicate monolayers were stained with anti-N antibodies (lower panels). **D.** Detection of NSs in mosquito cells infected with rMP12:S-Swap. Cells were infected and stained with anti-NSs (green) and anti-tubulin (red) antibodies as above.

A characteristic feature of RVFV NSs protein is that it forms ribbon-like structures in the nuclei of infected cells [30]. As the data shown in Figure 3 indicated that there was an augmented expression of NSs in rMP12:S-Swap-infected cells, we examined the effect of increased levels of NSs on cellular localisation and filament formation by immunofluorescent imaging. Vero-E6, C6/36, U4.4 or Ae cells were infected with rMP12 or rMP12:S-Swap at an MOI of 1, fixed at 12, 24 or 48 h p.i., and stained with anti-NSs and anti-tubulin antibodies (Figure 4). In rMP12-infected Vero-E6 cells thin NSs filaments in the nuclei were seen from 12 h p.i., and there was little cytoplasmic staining (Figure 4A). By contrast, in rMP12:S-Swap-infected cells, the nuclear filaments appeared thicker, and there was more abundant cytoplasmic staining of NSs, particularly by 24 h p.i. (Figure 4A). The average thickness of the filaments was determined by measuring the width of about 50 structures derived from each virus; those of rMP12 averaged 1.19 µm while those produced by rMP12:S-Swap averaged 2.21 µm (Figure 4B). The difference was statistically significant (Student's t-test, p<0.0001).

NSs protein was detected in the nuclei of *A. albopictus* C6/36 cells infected with both rMP12 or rMP12:S-Swap, though not in the form of filaments as seen in Vero-E6 cells (Figure 4C, upper panel, and 4D). Staining appeared granular or as large aggregates. In the majority of rMP12-infected U4.4 or Ae cells no NSs was detected, but in rMP12:S-Swap-infected cells aggregates of NSs were observed in the nuclei (Figure 4D). The infection status of the rMP12-infected insect cell monolayers was monitored in duplicate cultures by staining with the anti-N antibody, which showed that by 48 h p.i. all cells in the monolayer were infected despite a lack of NSs staining (Figure 4C, lower panel).

### Persistence of recombinant viruses in mosquito cell lines

**Figure 5:**
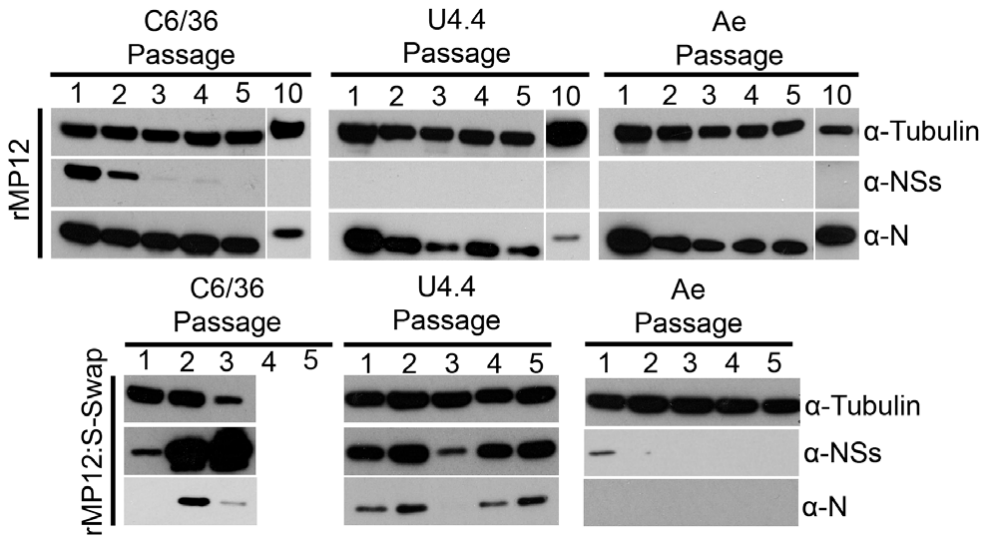
Serial passage of mosquito cells infected with rMP12 or rMP12:S-Swap. *A. albopictus* C6/36, U4.4 cells or *A. aegypti* Ae were infected with rMP12 or rMP12:S-Swap at a MOI of 0.01. Cell monolayers were passaged (split ratio 1∶5) every 5–7 days (when 100% confluency was observed). Cell extracts were prepared from each passage, proteins fractionated SDS-PAGE, transferred to a membrane, and blots probed with anti-N, anti-NSs and anti-tubulin antibodies as indicated. C3/36 cells infected with rMP12:S-Swap died after passage 3.

As previously reported by Léger *et al.* [31] infection of mosquito cells (in this case *A. albopictus* U4.4 and *A. aegypti* Aag2 cells) with a virulent strain of RVFV leads to the establishment of a persistent infection, though differences in the expression of NSs were observed in the different cell types. In agreement with their findings we observed suppression of NSs production in U4.4 cells and in the *Aedes egypti* derived Ae cells, and the cells became persistently infected as shown by detection of N protein (Figure 5) and of released virus by plaque assay (data not shown) during sequential passage. The NSs protein was readily detected in rMP12-infected C6/36 cells for two passages, after which the abundance of the protein decreased to undetectable levels by passage 5 through to passage 10 during the establishment of persistence (Figure 5). The S RNA segment of virus released from the cells at passages 5 and 10 was amplified by RT-PCR and the nucleotide sequence determined. In all cases no mutations in the S segment were observed and the NSs ORF was intact (data not shown). Different effects were noted in cells infected with rMP12:S-Swap. C6/36 cells showed dramatic over-expression of NSs during 3 passages, after which the cells detached from the substrate and were killed by the infection. In U4.4 cells NSs was readily detected during establishment of persistent infection, but the cells showed cytopathic effects that were widespread at passage 3. However, sufficient viable cells were harvested to allow continued passage, but maintenance of the cells beyond passage 5 was not possible due to the death of all remaining cells. NSs was detectable at each passage (though not as much as in C6/36 cells) with the decrease seen at passage 3 due to a reduced number of cells available to assay. Similarly, the apparent lack of N at passage 3 was due to the reduction in the amount of cells to assay; N was detectable at both passages 4 and 5. Ae cells did not facilitate rMP12:S-Swap persistence in the monolayers and the virus was lost after one passage, evidenced by the lack of N or NSs in cell lysates (Figure 5).

### NSs-induced inhibition of mammalian cell protein and RNA synthesis

**Figure 6:**
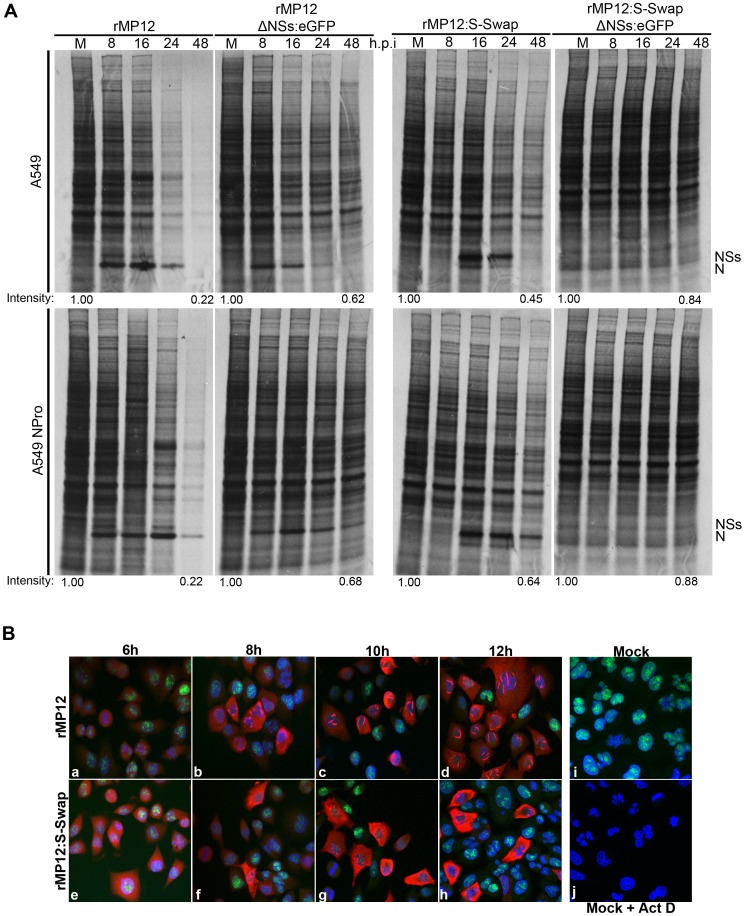
Inhibition of host cell protein and RNA synthesis. A. Protein synthesis. A549 or A549 NPro cells were infected with rMP12, rMP12:S-Swap, rMP12ΔNSs:eGFP or rMP12:S-SwapΔNSs:eGFP at a MOI of 3. Cells were labelled with 30 µCi [^35^S] methionine/cysteine for 2 h at the time points indicated, and cell extracts were fractionated by SDS-PAGE. The positions of the viral N and NSs proteins are shown. Total lane intensities were measured by densitometry and compared to the mock-infected sample for each virus time course as indicated. **B.** RNA synthesis. A549 cells were infected with rMP12 (panels a to d) or rMP12:S-Swap (panels e to h) at a MOI of 3. One hour prior to the time points indicated the uridine analogue 5-ethynyl uridine (EU) was added to the medium and then cells were fixed in 4% formaldehyde. Cells were processed using Click-iT RNA AF488 Imaging Kit (newly synthesised RNA stains green), and then reacted with anti-NSs antibodies and secondary goat anti-rabbit Alexa Fluor 633 antibody (red). As controls, mock-infected cells were left untreated (i) or treated with actinomycin D (Act D) at 5 µg/ml for 1 h prior to 5-EU treatment (j).

One of the mechanisms of action of NSs in mammalian cells is a global inhibition of host protein synthesis, mediated at both the transcriptional level through the degradation of the p62 and the sequestration of the p44 subunits of TFIIH [8], [9], and the translational level by the degradation of the cellular kinase PKR [10], [11]. We therefore investigated if there was a difference in protein synthesis inhibition in recombinant virus-infected cells. A549 (interferon competent) or A549 NPro (interferon incompetent) cells were infected or mock infected with recombinant viruses at a MOI of 3. At the time points indicated, the cells were labelled with [^35^S] methionine/cysteine for two hours, and cell lysates separated by SDS-PAGE (Figure 6). In rMP12-infected cells the N protein was identified at 8 h p.i with a faint NSs band visible above, and by 24 h p.i. there was a marked reduction in incorporation of radiolabel into host proteins compared to mock-infected cells. The decrease was even more pronounced at 48 h p.i. in both A549 and A549 NPro cell lines. In rMP12:S-Swap-infected cells, even though there was considerably greater synthesis of NSs, there was no increase in host cell protein synthesis inhibition in comparison to rMP12-infected A549 or A549 NPro cells. In fact, the degree of inhibition assessed by densitometry of the lanes was less than in rMP12 infected cells. As a control, and to show that host protein synthesis inhibition was largely mediated by the presence of NSs, rMP12ΔNSs:eGFP and rMP12:S-SwapΔNSs:eGFP cell lysates were also examined. In all cases, the incorporation of radiolabel over the 48 h duration of the experiment was greater than in the cells infected with NSs-expressing viruses.

As protein synthesis reflects the relative abundance of cellular mRNAs within the cell, we also determined the level of transcriptional activity in infected cells. A549 cells were infected with recombinant viruses at MOI of 3 or mock-infected. A dish of mock-infected cells was also treated with actinomycin D (Act-D) as a positive control for transcriptional inhibition. One hour before the time points indicated the uridine analogue 5-ethynyl uridine (EU) was added to the medium to enable RNA synthesized within this hour period to be detected by click chemistry [32], [33], and then cells were stained with anti-NSs antibody (Figure 6B, panels a-h). In rMP12-infected cells punctate nuclear NSs staining was detected by 8 h p.i. (panel b), and by 12 h p.i. the characteristic RVFV NSs nuclear filaments were seen in the nuclei of infected cells (panel d). In rMP12:S-Swap-infected cells, NSs staining was also detected by 8 h p.i. (panel g), though the staining was predominantly cytoplasmic rather than nuclear. Irregular and thicker nuclear filaments were detected in rMP12:S-Swap-infected cell nuclei from 10 h p.i. onwards along with an increased and more intense cytoplasmic staining of NSs. In the early stages of infection (6 h p.i.) RNA synthesis could be detected in the nuclei of cells infected by either virus. However, RNA staining was markedly reduced when NSs nuclear filaments were first observed, similar to that the effect seen when cells were treated with Act D (Figure 6B, panel j). Thus rMP12:S-Swap inhibited host RNA synthesis similarly to rMP12.

**Figure 7:**
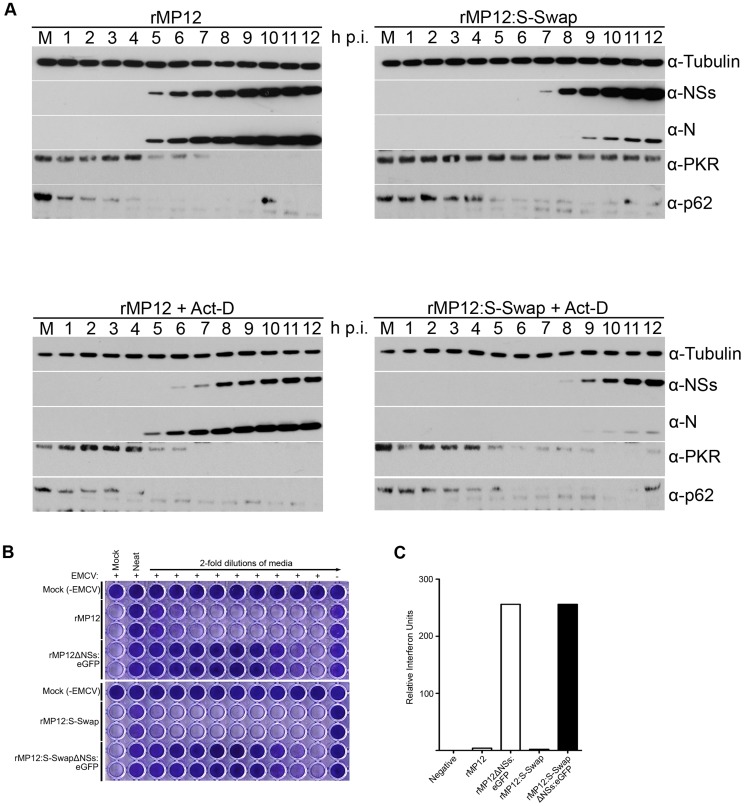
Effect of RVFV NSs on host cell factors. **A.** Effect on PKR and p62. A549 cells or A549 cells treated with 5 µg/ml actinomycin D were infected with rMP12 or rMP12:S-Swap at a MOI of 3, or mock-infected. Cell extracts were prepared at the time points indicated, proteins fractionated by SDS-PAGE, and blots probed with anti-N, anti-NSs, anti-PKR, anti-p62, and anti-tubulin antibodies as indicated. **B.** Induction of interferon. A549 cells were infected with rMP12, rMP12ΔNSs:eGFP, rMP12:S-Swap or rMP12:S-SwapΔNSs:eGFP at MOI of 5, and supernatants harvested at 18 h p.i. After UV treatment, 2-fold dilutions were applied to A549-NPro cells for 24 h, before infection with EMCV. Monolayers were stained with Giesma after a further 96 h. **C.** The amount of IFN produced is expressed as relative IFN units (RIU), defined as RIU = 2^N^ where N = the number of two-fold dilutions of the supernatants that protected the reporter cells from EMCV challenge.

Next we examined fate of PKR and p62 in A549 cells infected with rMP12 or rMP12:S-Swap at a MOI of 3 (Figure 7A, upper panels). At this multiplicity, N and NSs proteins were first detected at 5 h p.i. in rMP12-infected cells. The amount of PKR detected started to decrease from 5 h p.i. and was undetectable by 9 h p.i. The amount of p62 present in the cell lysate was also depleted starting at 3 h p.i. and was undetectable by 5 h p.i. In rMP12:S-Swap-infected A549 cells, NSs was first detected at 7 h p.i. and N at 9 h p.i. Unexpectedly, there was little decrease in the amount of PKR present in the cells over the 12-hour time course (Figure 7A). By contrast, a decrease in p62 was noted by 5 h p.i. Thus in both rMP12- and rMP12:S-Swap-infected cells degradation of p62 was observed just before NSs was readily detected.

Act-D treatment of infected A549 cells had a small effect on N and NSs expression by both viruses compared to the levels observed in the respective untreated cells (Figure 7A, lower panels). However, more noticeable was that PKR degradation occurred in rMP12:S-Swap-infected Act-D treated A549 cells from 4 h p.i. whereas no degradation was seen in uninfected control cells. Loss of p62 was observed by 3–4 h p.i. for both viruses, suggesting that ActD treatment induced a decrease in p62 faster than NSs (Figure 7A).

Lastly, we examined the production of interferon in cells infected with the recombinant viruses at a MOI of 5 after 18 hours using a biological assay [34]. A549-NPro cells were treated with UV-inactivated medium from infected cells before being infected with the interferon-sensitive encephalomyocarditis virus (EMCV). Subsequently the cells were examined for the protection or not from EMCV-induced cytopathic effects. rMP12 induced approximately twice as much interferon as rMP12:S-Swap, whereas both of the NSs deletant viruses generated approximately 64 times as much interferon (Figure 7B and 7C).

### Analysis of viral RNA in infected cells and virus particles

**Figure 8:**
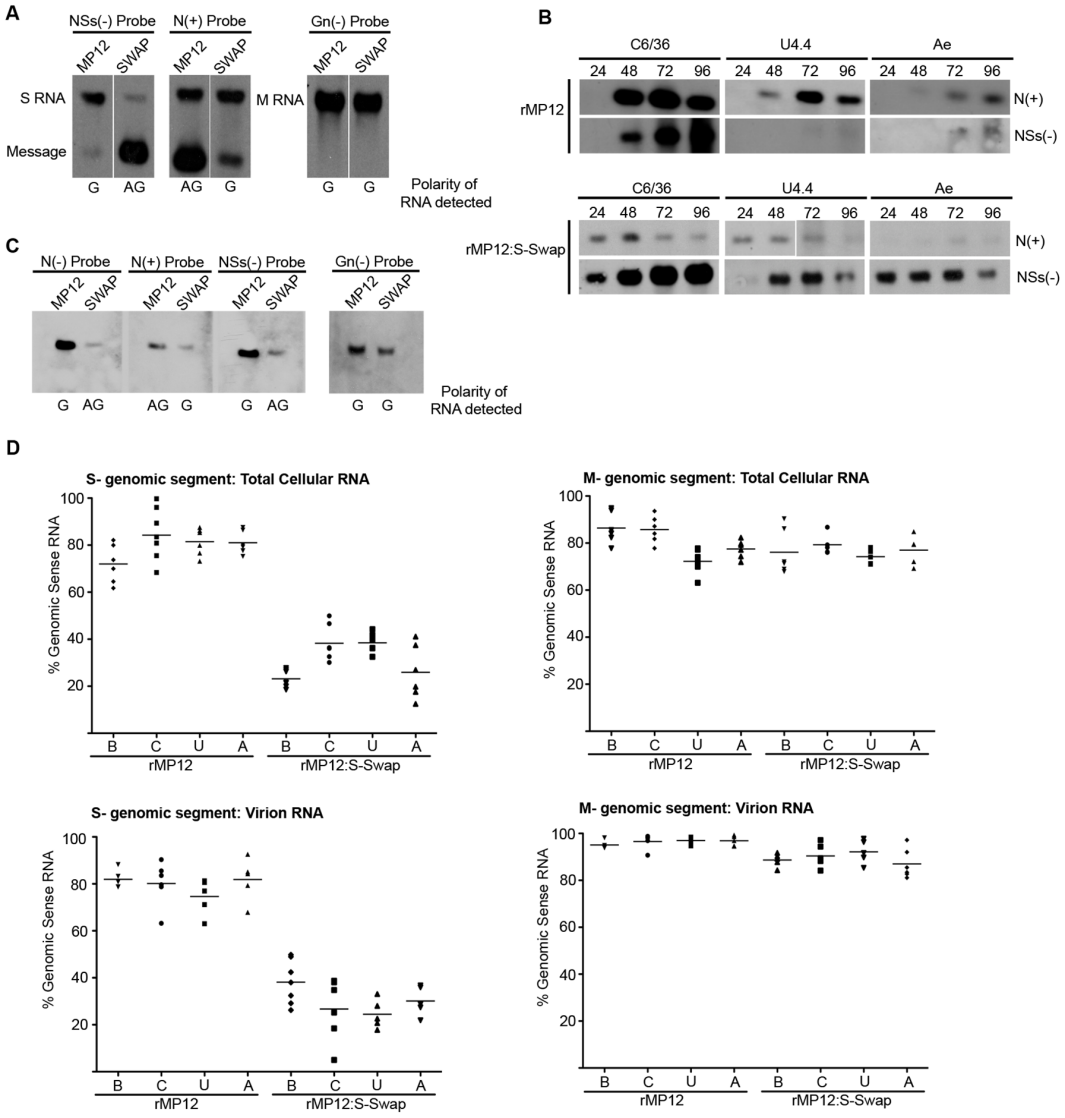
Analysis of viral RNAs. A. Intracellular RNAs in infected BHK-21 cells. Cells were infected with rMP12 or rMP12:S-Swap (MOI of 1), and total cell RNA isolated at 48 h p.i. Northern blotting was performed using DIG-labelled probes complementary to the N, NSs, and Gn coding regions in the viral genomic (−) sense RNA and the N coding region in the viral anti-genomic (+) sense RNA. The polarity of the RNA detected by each probe is indicated below the blot: G, genomic sense RNA and AG, anti-genomic sense RNA, defined by the sequence of the 3′/5′ untranslated regions. **B.** S segment derived mRNAs produced in mosquito cells. *A. albopictus* C6/36, U4.4 cells or *A. aegypti* Ae were infected with rMP12 or rMP12:S-Swap at MOI of 1, and total cellular RNA extracted at the indicated times p.i. Northern blotting was performed using the N(+) and NSs(−) probes. **C.** Analysis of RNA packaged into virions. RNA was extracted purified rMP12 or rMP12:S-Swap grown in BHK-21 cells, and Northern blotting performed with the probes as detailed in (A) above. **D.** Quantitative RT-PCR analysis of viral RNAs. Total cellular RNA (upper panels) or RNA in purified virus particles (lower panels) of rMP12 or rMP12:S-Swap was analysed using probes specific form the S or M segments as described in Methods. The results are presented as the percentage of genomic RNA species compared to the total RNA of the same segment. Cell line abbreviations: B, BHK-21; C, C6/36; U, U4.4; and A, Ae.

Viral RNAs present in infected cells were examined to ascertain if the difference in levels of N and NSs protein synthesised by rMP12- or rMP12S:Swap was mediated at the transcriptional level. Northern blot analysis of total cellular RNA from infected BHK-21 cells was undertaken using strand-specific digoxigenin-labelled RNA probes for N, NSs and Gn coding regions (Figure 8A). The NSs(−) probe detected full-length S RNA (1690 nt) in both rMP12- and rMP12:S-Swap-infected cells, corresponding to genomic and anti-genomic RNA respectively. There appeared less anti-genomic S RNA in rMP12:S-Swap infected cells. The subgenomic NSs mRNA (approximately 900 nt) was also detected with the NSs(−) probe, and was more abundant in rMP12:S-Swap-infected cell lysates than in cells infected with the parental virus rMP12. Hybridisation with the N(+) probe detected the N mRNA (approximately 850 nt) in cells infected with either rMP12 or rMP12:S-Swap, however to a much greater extent in rMP12-infected cells. The N(+) probe also detected rMP12 S anti-genomic RNA and rMP12:S-Swap genomic RNA species. No detectable difference in the amount of M genomic RNA was detected between the rMP12 or rMP12:S-Swap intracellular RNA samples using the Gn(−) probe (Figure 8A).

A similar approach was used to examine the N and NSs subgenomic mRNAs produced in rMP12- or rMP12:S-Swap-infected C6/36, U4.4 or Ae total cell RNA extracts over a 96 h time course of infection (Figure 8B). N and NSs mRNAs were readily detected in C6/36 cells infected with either virus. In rMP12-infected U4.4 or Ae cells, NSs mRNA was barely detectable, if at all, reflecting earlier data shown in Figure 3C that no NSs protein was made during this period. When rMP12:S-Swap-infected total cell RNA extracts were examined, NSs message was strongly detected in these cells, whereas the signal for N mRNA was much weaker (Figure 8B).

The RNA packaged into rMP12 and rMP12:S-Swap virus particles was also examined by Northern blotting. For rMP12 virion RNA, the N(−) and NSs(−) probes detected S genomic RNA and the N(+) probe also detected a lesser amount of packaged antigenomic RNA. Conversely, when rMP12:S-Swap derived virion RNA was analysed, the N(−) and NSs(−) probes detected antigenomic S RNA and the N(+) probe detected genomic sense RNA, which was present in the lesser amounts. As a control the genomic M RNA was analysed by hybridisation with the Gn(−) probe and showed similar amounts of virion RNA were loaded on the gel from rMP12 or rMP12:S-Swap virus particles (Figure 8C).

The above results suggested that there were different ratios of genomic to antigenomic S RNA present in infected cells and packaged into virions. As it is difficult to quantify levels of RNA in infected cells by Northern blotting, we re-examined the same RNA samples using strand-specific qRT-PCR (Figure 8D). (Optimisation of the assay is presented in Supplementary Figures S1 and S2, and Table S2). Viral genomic or antigenomic S- or M-segment RNA was selectively amplified with segment-specific tagged oligonucleotides as described above. In rMP12-infected BHK-21 cells the mean proportion of the two S RNA species was 72.0% genomic RNA and 28.0% antigenomic RNA. However, when the rMP12:S-Swap infected-cell BHK-21 RNA was examined the mean proportion of S genomic RNA was reduced to 23.2% of the S RNA present in the cell. In contrast there was no difference in the mean proportions of M segment RNA in either rMP12 or rMP12:S-Swap infected BHK-21 cells, with 98.9% or 99.1% genomic RNA and 1.1% or 0.9% antigenomic RNA recorded respectively (Figure 8D).

Next, we investigated whether the difference in the ratio of genomic to antigenomic S RNA seen in rMP12- and rMP12:S-Swap-infected cells was reflected in the RNA packaged into progeny virions. For rMP12 grown in BHK-21 cells, we observed mean values of 82% genomic and 18% anti-genomic S RNA, whereas for rMP12:S-Swap the mean values were 43.7% genomic and 56.3% anti-genomic RNA. In contrast there was no significant difference in the proportions of packaged M segment RNAs between the two viruses: 98.6%∶1.4% genomic∶antigenomic for rMP12 and 98.1%∶1.9% genomic∶antigenomic for rMP12:S-Swap. We also investigated the RNA species packaged when the viruses were grown in mosquito cells, and saw similar patterns to that described for BHK-21 cell grown viruses (Figure 8D).

We analysed these data to determine whether the differences in RNA populations in total cell RNA and virion RNA were statistically significant. Five independent RNA preparations were analysed for each cell type, and the mean percentage of genomic RNA (defined by the 3′ genomic UTR sequence of rMP12) was calculated. The composition of the RNA population was analysed using a one-way ANOVA with Tukey's multiple comparison post-test; significance was set at p<0.05. The results are summarised in Table 1 (raw data can be found in Supplementary Table S3 and S4). In all cell lines tested there was a significant difference in the percentage of genomic S segment RNA produced within the infected cell and between the relative amounts of genomic S segment RNA packaged in virus particles when comparing rMP12 to rMP12:S-Swap. Conversely, there was no significant difference in the proportion of genomic M segment RNA present in either rMP12- or rMP12:S-Swap total cellular RNA samples or virion RNA. This suggests that changes in proportions of S RNA species present in infected-cells or packaged into a virion were associated with the reconfigured S-RNA. However, there was no significant difference in the proportion of genomic RNA present in total cell RNA extracts compared to that detected in virions: that is, whatever polarity of viral RNA segment that was most abundant within the total cell RNA population was also the prevalent species packaged in virus particles (Table 1).

**Table 1:** Summary of qRT-PCR analysis.

	S Segment			
Samples Compared	C6/36	U4.4	Ae	BHK
**rMP12 Virion vs. rMP12:S-Swap Virion**	YES^a^	YES	YES	YES
**rMP12 Virion vs. MP12 Total**	NO^b^	NO	NO	NO
**rMP12:S-Swap Virion vs. rMP12:S-Swap Total**	NO	NO	NO	NO
**rMP12 Total vs. rMP12:S-Swap Total**	YES	YES	YES	YES

	M Segment			
Samples Compared	C6/36	U4.4	Ae	BHK
**rMP12 Virion vs. rMP12:S-Swap Virion**	NO	NO	NO	NO
**rMP12 Total vs. rMP12:S-Swap Total**	NO	NO	NO	NO

a , significantly different, p<0.05 (see Methods for details).

b , not significantly different,

## Discussion

The ambisense coding strategy adopted by some members of the *Bunyaviridae* and the *Arenaviridae* is an intriguing way of regulating temporal gene expression [22], [23]. The ambisense RVFV S segment encodes the N and NSs proteins that are translated from individual subgenomic mRNAs. The N mRNA is transcribed from the genomic S segment whereas the NSs mRNA is transcribed from the antigenome S segment (Figure 1A). In this study, we investigated the consequences of swapping the N and NSs coding sequences on the S segment by directly substituting the N ORF into the NSs locus and the NSs ORF into the N locus (Figure 1). We were able to recover, by reverse genetics, a virus that contained the modified S segment, designated rMP12:S-Swap. This strategy left the 3′, 5′, and intergenic untranslated regions untouched, though it is possible that cis-acting signals spanning untranslated and coding sequences (e.g. for packaging or transcription termination) could be disrupted. The replication kinetics of rMP12:S-Swap, the parental virus rMP12, and recombinant viruses in which the NSs ORF was replaced with that of the eGFP (rMP12ΔNSs:eGFP and rMP12:S-SwapΔNSs:eGFP) were compared in a range of mammalian and insect cell lines. Viruses that contained the reconfigured genomic S segment gave titres 10- to 1000-fold lower than the corresponding viruses with the normal S segment configuration (rMP12 or rMP12ΔNSs:eGFP) in all cell lines tested (Figure 2A, 2C). Reduced growth appeared to be correlated with the marked reduction in the amount of N protein produced over 48 h in rMP12:S-Swap- and rMP12:S-SwapΔNSs:eGFP-infected cells compared to that of the parental viruses. Although synthesis of the Gn glycoprotein was less in reconfigured viruses early in infection, this appeared to reflect their slower replication kinetics. At 18 and 24 h p.i. the titre of rMP12:S-Swap was approximately 1000-fold less than rMP12 and Gn was weakly detected. However, by 48 h p.i., there was only a 10-fold difference between the virus titres, and much less difference in the intensity of Gn bands (Figure 3A).

Initially, it was thought that the decrease in the amount of N produced in rMP12:S-Swap infected cells could have been due to the increased and earlier expression of the NSs protein. Previous work in our laboratory had shown that the NSs protein of RVFV could inhibit a *Renilla*-based minigenome in a dose dependent manner [14], implying a regulatory role of NSs in RVFV polymerase activity. However, the NSs-deleted virus rMP12:S-SwapΔNSs:eGFP also showed a reduced amount of N protein (Figure 3A) indicating that NSs was not involved.

A direct visual comparison of the differential protein expression between the parental and reconfigured viruses was provided by observing eGFP fluorescence in rMP12ΔNSs:eGFP- and rMP12:S-SwapΔNSs:eGFP-infected BHK-21 monolayers: eGFP fluorescence was more intense in rMP12:S-SwapΔNSs:eGFP- compared to that of rMP12ΔNSs:eGFP-infected cells. This indicates that the authentic MP12 ‘N promoter’ sequence (antigenomic 5′ UTR) driving eGFP expression in rMP12:S-SwapΔNSs:eGFP is much stronger than that of the authentic MP12 ‘NSs promoter’ sequence (genomic 5′ UTR). Earlier studies have reported that the UTRs on different segments have different promoter strengths [25], [35]–[40], and though a direct comparison of N and NSs promoter strengths was not obtained, it was observed that NSs mRNA levels were significantly less than those of N mRNA in infected cells. Thus differences between promoter strengths on genomic and antigenomic RNAs are likely to be responsible for the difference in N protein levels observed between parental and reconfigured viruses, rather than any specific inhibition of N protein production.

Previous reports have suggested potential roles for the nonstructural proteins of bunyaviruses in the maintenance of a virus in the natural infectious cycle, persistence in infected insect cells, and dissemination of a virus throughout an infected mosquito [29], [31], [41]–[45]. Therefore we investigated how the differential expression of NSs affected the ability of the virus to replicate in cultured mosquito cells. We found that NSs protein was readily detectable in rMP12-infected *A. albopictus* C6/36 cells, as was the NSs subgenomic mRNA (Figure 8B) but neither NSs nor eGFP could be detected in *A. albopictus* U4.4 or *A. aegypti* Ae cells that had been infected with rMP12 or rMP12ΔNSs:eGFP respectively. Similarly NSs mRNA could not be detected by Northern blotting (Figure 8B). This is in line with published results showing that in U4.4 and another *A. aegypti* cell line, Aag2, NSs expression is down regulated at the transcriptional level by a Dicer-2 mediated RNAi response. This RNAi response, characterised by 21-nt long viRNAs, is non-functional in C6/36 cells, due to a deletion in the *dicer-2* gene, and may explain why NSs can be expressed during infection (Figure 3C) [31], [46]. The difference in RNAi responses is presumably the reason why rMP12 grows to higher titres in C6/36 cells (Figure 2C) compared to other mosquito lines. Unexpectedly, there was no detectable eGFP expression in C6/36 cells infected with rMP12ΔNSs:eGFP. Despite not having a functional Dicer-2 protein, C6/36 cells are able to mount a Piwi-mediated RNAi response [47]–[49], characterised by 24–30-nt long viRNAs, and in the absence of NSs expression (as in rMP12ΔNSs:eGFP-infected cells) the Piwi-pathway may be sufficient to degrade the eGFP-mRNA [31].

When the three insect cell lines were infected with either rMP12:S-Swap or rMP12:S-SwapΔNSs:eGFP, the NSs protein was detected in cell lysates by western blotting or eGFP fluorescence was observed microscopically as appropriate. By contrast, N protein levels were much reduced, particularly in U4.4 and Ae cells, compared to infection with parental viruses (Figure 3C, 3D). Based on the western and northern blot data, it is not possible to determine whether RNAi contributes to the down-regulation of N observed in U4.4 and Ae cells, or whether the lower amounts of N just reflect the weaker promoter driving transcription of its mRNA. The study by Léger *et al.* [31] revealed hot spots for viRNA targets amongst the genome segments, one of which appears to be in the antigenomic sense NSs region that could degrade its mRNA. It would be instructive to compare the profiles of small RNAs produced in mosquito cells infected with rMP12 and rMP12:S-Swap to investigate whether the pattern of S segment viRNA hot spots differs with the reconfigured S RNA.

Down-regulation of NSs by RNAi is thought necessary for RVFV to establish persistence in mosquito cells, as a persistent infection could not be established by the ZH548 strain in Dicer-2 deficient C6/36 cells [31]. Accordingly, persistent infections of U4.4 cells and Ae cells were readily established by rMP12 and no NSs protein could be detected. However, we were able also to establish persistent infection of C6/36 cells with rMP12; NSs levels markedly decreased at each passage of the cells, perhaps mediated by piRNAs. The MP12 strain contains mutations on all three segments that attenuate replication in mammalian cells [50]; it would be of interest to determine which mutations are associated with the ability to establish persistence in *A. albopictus* C6/36 cells. However, when C6/36 cells were infected with rMP12:S-Swap, western blotting showed that NSs was hyper-expressed, and many cells detached from the monolayer during infection. By passage 3 all the cells had been killed by the infection and hence persistence could not be maintained. U4.4 cells also showed similar evidence of cytopathic effect, and cells did not survive beyond passage 5.

Thus we suggest that the RNAi response is able to modulate though not ablate NSs expression. Taken together, the data indicate that high levels NSs are toxic to mosquito cells, perhaps through a comparable transcriptional inhibition as occurs in mammalian cells. The RNAi system in U4.4 cells normally represses NSs expression almost completely when infected with virulent or attenuated strains; initially, it seems able to control NSs expression sufficiently to enable cell survival when infected with rMP12:S-Swap. However, accumulation of NSs after a few passages resulted in cell death. In this regard analysis of viRNA populations in U4.4 cells persistently infected with rMP12:S-Swap would be of interest. We were unable to establish persistence in Ae cells infected with rMP12:S-Swap. It is possible that the RNAi response is even more robust in these cells compared to U4.4. cells, and as no cytopathic effects were seen following infection with rMP12:S-Swap, we presume that the amount of N protein produced was insufficient to enable a productive replication cycle to begin.

The NSs protein characteristically forms nuclear filaments in infected mammalian cells [30], [51], [52], and filament formation is independent of other viral proteins [53]. A consequence of the increased expression of NSs by rMP12:S-Swap was that the filaments were much thicker than those produced by rMP12, and in addition there was an increased abundance of NSs protein in the cytoplasm of rMP12:S-Swap-infected Vero-E6 cells at later times during infection. In contrast, although NSs was detected in the nuclei of infected mosquito cells, no filament formation was observed. Interestingly, the NSs protein expressed by the virulent ZH548 strain of RVFV did form intranuclear filaments in infected *A. albopictus* C6/36 cells [31] whereas the NSs of the attenuated MP12 strain appeared to form aggregates (Figure 4C). There is a single amino acid difference between the two NSs proteins (residue 160 is valine in ZH548 and alanine in MP12; [50]) but in the absence of structural information on NSs it is not possible to understand how this residue affects filament formation.

In mammalian cells a major function of the NSs protein is to elicit global inhibition of host cell transcription, mediated by effects on transcription factor TFIIH subunits [8], [9]. We therefore examined the effect that excess NSs (produced during rMP12:S-Swap infection) had on the ability of the virus to shut off protein or RNA synthesis (Figure 6). Surprisingly, there was no difference in the ability of rMP12:S-Swap to abrogate protein synthesis compared to the parental virus, despite a very obvious over-expression of the NSs protein at 16 and 24 h p.i. in rMP12:S-Swap-infected cell lines. This suggests that the amount of NSs produced in an rMP12-infected cell is already at a saturating level and that any excess NSs produced (such as during rMP12:S-Swap infection) cannot increase the rate at which global host cell protein synthesis inhibition occurs (Figure 6A). Inhibition of RNA synthesis was strongly correlated with formation of nuclear NSs filaments. A marked reduction in RNA synthesis was seen in infected-cells upon detection of nuclear localised NSs staining, indicating that NSs expressed by rMP12:S-Swap was able to inhibit host cell RNA synthesis in the same manner as that of rMP12, in agreement with previous reports [9], [12].

In both rMP12- and rMP12:S-Swap-infected cells we observed rapid depletion of p62, the timing of which correlated with expression of NSs. However, there was no appreciable reduction in the level of PKR in the presence of enhanced NSs expression in rMP12:S-Swap-infected cells. PKR degradation in RVFV-infected cells is mediated by NSs via the ubiquitin cellular proteasomal pathway [10], [11]. Therefore, our data suggest that in rMP12:S-Swap-infected cells this pathway could be blocked somehow, perhaps due to an overwhelming amount of NSs being present in the cytoplasmic compartment of the cell (visualised in Figure 4A). Our data are also consistent with a recent report that p62 and PKR are degraded in virus-infected cells independently of one another [12]. In this paper the authors described a recombinant virus rMP12-NSsR173A, which expressed NSs containing substitution of amino acid 173, that inhibited host RNA synthesis and IFN-β gene expression, but was unable to degrade PKR. Furthermore, the mutant NSs filaments appeared different from those of parental NSs, forming an irregular mosaic-like pattern. The authors suggested that the PKR degradation might involve a domain in NSs containing residue 173. The phenotype of rMP12:S-Swap is remarkably similar to that of rMP12-NSsR173A, and perhaps the over-expression of NSs by the former virus sterically inhibits the interaction needed for PKR degradation. Alternatively, the delay in inhibition of transcription observed in rMP12:S-Swap infected cells may allow sufficient expression of a factor that prevents PKR degradation once NSs is expressed.

Finally, to assess whether the changes in NSs protein expression were represented at the transcriptional level we examined the production of viral RNAs in virus-infected cells. There was substantially more NSs mRNA transcribed from the viral genomic S RNA of rMP12:S-Swap than from the viral anti-genomic S RNA of rMP12 suggesting that this is basis for the increased NSs protein production seen during rMP12:S-Swap infection (Figure 8A). This effect was particularly evident when examining the subgenomic N and NSs mRNAs produced during infection of insect cells. As discussed earlier, it appears that the insect RNAi response targets the NSs mRNA of rMP12 and reduces the cellular concentration of any mRNA originating from that locus: NSs in rMP12 and N in rMP12:S-Swap (Figure 8B).

Importantly, there also appeared to be a substantial difference in the relative abundance and polarity of RNA packaged into progeny virions between the two viruses. rMP12 packages more genomic than antigenomic S RNA whereas for rMP12:S-Swap the opposite was apparent. This indicates that the panhandle structure formed by the 3′ and 5′ termini of each RNA segment is not the only prerequisite for the packaging of specific polarities of viral RNA, as the UTRs of both viruses have remained unchanged [54], [55]. It is possible that a packaging signal (RNA sequence or structure) resides within the anti-sense N coding sequence which determines what particular polarity of RNA is incorporated into a virus particle. On the other hand, as the population of RNAs, be it genome or antigenome, present in the infected cell reflected that packaged into virions, there may not be a selective packaging mechanism for one polarity of RNA over another, but rather it is a stochastic mechanism that packages the most abundant viral RNA species into the virus particle. Additional work is underway to investigate polarity-specific packaging further.

In conclusion, by transposing the relative orientations of N and NSs ORFs on the RVFV S genome segment, we have generated a virus whose growth is attenuated in cell culture and whose replication is cytotoxic in cultured mosquito cells. It would be of interest to assess whether rMP12:S-Swap or derivatives thereof could be useful for vaccine development. For instance, a version expressing a marker epitope in place of NSs would likely be attenuated in animals and capable of inducing protective immunity, as demonstrated for other NSs-deleted viruses [15], [16], and antibodies to the highly-expressed epitope could be useful in differentiating infected from vaccinated animals (DIVA). Alternatively, a protective antigen from another pathogen could be over-expressed instead of NSs to create a divalent vaccine. On the other hand, if over-expression of NSs results in death of an infected mosquito, this feature might be exploited to generate non-transmissible live vaccines. Such possibilities warrant further investigation.

## Materials and Methods

### Cells and viruses

Vero-E6, A549 and A549-NPro [56] cells were grown in Dulbecco's modified Eagle's medium (DMEM) supplemented with 10% fetal calf serum (FCS). BSR-T7/5 cells [57], which stably express T7 RNA polymerase, were grown in Glasgow minimal essential medium (GMEM) supplemented with 10% FCS, 10% tryptose phosphate broth (TPB) and 1 mg/ml G418. BHK-21 cells were grown in GMEM supplemented with 10% newborn calf serum (NCS) and 10% TPB. All mammalian cell lines were grown at 37°C with 5% CO_2_ unless otherwise stated.

*Aedes albopictus* C6/36 and U4.4 and *Aedes aegypti* Ae cells were grown at 28°C in Leibovitz 15 medium (Gibco) supplemented with 10% fetal calf serum (FBS) and 10% tryptose phosphate broth, as described previously [29].

All experiments with infectious virus were conducted under BSL-3 conditions. Stocks of recombinant viruses were grown in BHK-21 cells at 33°C by infecting at a multiplicity of infection (MOI) of 0.01 and harvesting the culture medium at 72 h post infection (p.i.).

### Plasmids

Plasmids for the recovery of RVFV have been described previously [27]. pTM1-L and pTM1-N contain the RVFV MP12 L and N ORFs under the control of T7 promoter and encephalomyocarditis virus internal ribosome entry site sequence; pTVT7-GS, pTVT7-GM and pTVT7-GL contain full-length cDNAs to the RVFV strain MP12 antigenome segments flanked by T7 promoter and hepatitis delta ribozyme sequences. Plasmid pTVT7-GS:S-Swap contains a full-length cDNA to the RVFV S segment in which the two S segment derived ORFs encoding the N and NSs proteins have been swapped, thereby creating a genomic S segment in which the N protein is expressed at the NSs locus and the NSs protein at the N locus. The swapped genomic segment was synthesised by GenScript USA Inc. and cloned into pTVT7 using *BbsI* restriction sites. The constructs pTVT7-GSΔNSs:eGFP or pTVT7-GS:S-SwapΔNSs:eGFP were created by replacing the NSs coding sequences with that of the enhanced green fluorescent protein (eGFP).

### Generation of recombinant viruses from cDNA

Recombinant RVFV were generated by transfecting 7×10^5^ BSR-T7/5 cells with 0.5 µg each pTM1-L and pTM1-N, and 1 µg each pTVT7-based plasmid expressing the viral antigenomic segments as appropriate, using 3 µl Lipofectamine 2000 (Invitrogen) per µg of DNA. After 5 to 7 days, when extensive cpe was observed, the virus-containing supernatants were collected, clarified by low speed centrifugation and stored at −80°C.

### Virus titration by plaque assay

BHK-21 cells were infected with serial dilutions of virus and incubated under an overlay consisting of GMEM supplemented with 2% NCS and 0.6% Avicel (FMC BioPolymer) at 37°C for 4 days. Cell monolayers were fixed with 4% paraformaldehyde and plaques were visualized by Giemsa staining.

### Western blotting

At different time points after infection cell lysates were prepared by the addition of 300 µl lysis buffer (100 mM Tris-HCl, pH 6.8; 4% SDS; 20% glycerol; 200 mM DTT, 0.2% bromophenol blue and 25 U/ml Benzonase (Novagen)) and proteins separated on a SDS-4–12% gradient polyacrylamide gel (Invitrogen). Proteins were transferred to a Hybond-C Extra membrane (Amersham), and the membrane was blocked by incubating in saturation buffer (PBS containing 5% dry milk and 0.1% Tween 20) for 1 h. The membrane was reacted with anti-N and anti-NSs polyclonal rabbit antibodies [14], an anti-Gn antibody (ProSci Inc.), an anti-eGFP antibody (Invitrogen) or an anti-tubulin monoclonal antibody (Sigma). This was followed by incubation with either horseradish peroxidase (HRP)-labelled anti-rabbit (Cell Signalling Technology) or anti-mouse (Sigma) antibodies. Visualization of detected proteins was achieved using SuperSignal WestPico chemiluminescent substrate (Pierce), followed by exposure to x-ray film.

### Metabolic radiolabeling

A549 or A549 NPro cells were infected with recombinant viruses at a MOI of 3. At the time points indicated, cells were incubated in starvation media lacking methionine for 30 min, washed, and then labelled with [^35^S] methionine/cysteine (30 µCi/ml; Amersham Pharmacia Biotech) for 2 h. Cell lysates were prepared by the addition of 300 µl lysis buffer (100 mM Tris-HCl, pH 6.8; 4% SDS; 20% glycerol; 200 mM DTT, 0.2% bromophenol blue and 25 U/ml Benzonase (Novagen)) and proteins separated by SDS-PAGE as described above. Visualisation of labelled proteins was achieved by exposure to a Storm840 Phospho-imager (Molecular Dynamics) or to X-ray film.

### Biological assay for interferon production

A549 cells in 35 mm dishes were infected with 5 PFU/cell of the recombinant viruses and incubated at 37°C for 18 h. The medium was removed and treated with UV light to inactivate any virus [34] and two-fold serial dilutions of the medium were applied to A549-NPro cells for 24 h. The cells were then infected with interferon-sensitive encephalomyocarditis virus (EMCV; 0.05 PFU/cell), and cells incubated for 4 days at 37°C. The cells were fixed with formaldehyde and stained with Giemsa stain to monitor development of CPE. The relative IFN units (RIU) are expressed as 2^N^ where N is the number of two-fold dilutions that protect the reporter cells.

### Immunofluorescent detection of host RNA synthesis

A549 cells were grown on glass cover-slips (13 mm diameter), and either infected with recombinant viruses, mock-infected or mock-infected and treated with Actinomycin D (5 µg/ml). The uridine analogue 5-ethynyl uridine (EU) was added to the culture medium for 1 h prior to harvest at the time points indicated, following the manufacturer's instructions (Click-iT RNA AF488 Imaging Kit; Invitrogen). Briefly, after 5-EU treatment, cells were permeabilized in 0.5% Triton X-100 in PBS and then incubated with the Click-iT reaction cocktail. Following treatment cells were reacted with RVFV anti-NSs primary (1∶250) and Alexa Fluor 633 anti-rabbit (1∶200) secondary antibodies. Localisation of the fluorescently labelled proteins and RNA was examined at various times post infection using a Zeiss LSM confocal microscope.

### Northern blotting and reverse transcription-PCR

BHK-21 cells were infected at a MOI of 1 and total cellular RNA was extracted at 48 h p.i. using TRIzol reagent (Invitrogen). Four micrograms of RNA was electrophoresed through a 1.2% agarose gel in TAE buffer [58] and then transferred to a positively charged nylon membrane (Roche). For the isolation of RNA from virus particles, RNA from 1×10^6^ virions of either rMP12 or rMP12:S-Swap virus stocks were extracted using the QIAamp Viral RNA Minikit (Qiagen). Equal volumes of eluted RNA were then electrophoresed through a 1.2% TAE gel and transferred to a positively charged nylon membrane as described above. The membranes were hybridized with digoxigenin-labelled RNA probes complementary to genome sense (−) or anti-genome sense (+) sequences representing Gn, N or NSs protein coding regions (150 ng of each probe); detection was carried out using a DIG Northern Starter Kit (Roche).

For reverse transcription-PCR (RT-PCR), 1 µg of total cellular RNA was mixed with a segment-specific oligonucleotide, 0.5 mM 4× dNTP mix (Promega), 40 U rRNasin (Promega) and 200 U M-MLV reverse transcriptase (Promega), and incubated at 42°C for 3 hours. The resulting cDNA was used in PCR reactions with primers as described in Results, and the products were visualized by agarose gel electrophoresis. Details of oligonucleotides used can be found in Table S1.

### Quantitative RT-PCR

Total cell RNA or virion RNA was extracted as described above, the resulting RNA quantified (Nanodrop Spectrophotometer; Thermo Scientific) and standardised to 2 ng/µl. Reverse transcription was performed as described above with an additional final incubation step of 90°C for 10 minutes to inactivate enzyme. Each reaction included a segment and strand-specific oligonucleotide (Table 2). A standardised control reaction was performed on in-vitro transcribed genomic and anti-genomic RNA to allow for normalisation of differences in experimental sensitivity. Details of oligonucleotides used in qPCR reactions can be found in Table 2.

**Table 2:** Oligonucleotides used in reverse transcription and qRT-PCR reactions.

Oligo	Sequence 5′-3′	Genome Position (Genomic Sense)
**S Segment**		
N FWD	AACTCTACGGGCATCAAACC	1539–1558^a^
N REV-TAG	GGCCGTCATGGTGGCGAATAAGAGCTTGCGATCCAGTT	1620–1639^a^
NSs FWD-TAG	GGCCGTCATGGTGGCGAATGGACTCCTTTGCTGGCTTAC	502–521^a^
NSs REV	GCACTGTACGTGAGCAACCT	597–616^a^
**M Segment**		
M FWD	CCGGTGCAACTTCAAAGAGT	10–29^b^
M REV	AGGCAGCAGCAGTCTCAAGT	90–109^b^
M FWD-TAG	GGCCGTCATGGTGGCGAATCCGGTGCAACTTCAAAGAGT	
M REV-TAG	GGCCGTCATGGTGGCGAATAGGCAGCAGCAGTCTCAAGT	
**TAG Oligo**	GGCCGTCATGGTGGCGAAT	

a From Rift Valley fever virus strain MP-12 segment S, DQ380154.

b From Rift Valley fever virus strain MP-12 segment M, DQ380208.

Tagged oligonucleotides were used for strand specific reverse transcription. The TAG sequence is underlined.

Quantitative PCR (qPCR) was carried out using MESA Blue qPCR MasterMix for SYBR Assay (Eurogentec) on a ABI 7300 platform (Applied Biosystems). Each reaction was performed in a 10 µl volume (8.4 µl qPCR MasterMix, 0.3 µl strand specific primer (10 µM) and 0.3 µl of TAG-primer (10 µM) and 1 µl cDNA template). Each reaction contained the opposing primer to the one used in the RT reaction and the TAG-primer (e.g. the rMP12 S genomic RNA RT-primer was N REV-TAG and the qPCR primers were N FWD and TAG-primer). The cycling parameters were 50°C for 2 minutes, 95°C for 10 minutes, followed by 40 cycles of 95°C for 15 s and 60°C for 60 s. A dissociation stage was included at the end of each reaction as a quality control step. A melt curve analysis at the end of each assay consisted of 95°C for 15 seconds, 60°C for 1 minute before increasing temperature to 95°C at 0.1°C per second. Each reaction was performed in triplicate with a mean Ct value calculated post assay. Each cell type and virus combination was tested a minimum of five times with unique infections and RNA preparations.

Ct values obtained for genome and antigenome assays were compared to determine the ratio of genome to antigenome in the original RNA preparation. Ct values were corrected using data from the segment specific standard curves. Differences in Ct values were calculated (genome Ct – antigenome Ct). The square root of the square of this number was calculated to remove any negative values. The relative amount difference between the two values was calculated using the formula x = e^(0.6931 y)^ where y is the difference in Ct values.

The calculated figure gives the relative amount difference between the genomic and antigenomic S or M segments in the original RNA sample. From this a percentage was be calculated (% genome = x/(x+1) ) which was used in all further statistics and figures. Graphs were produced, and one-way ANOVA with Tukey's multiple comparison post-test were performed, using GraphPad Prism version 5.0a for Mac OS X (GraphPad Software, La Jolla California USA, www.graphpad.com). Significance was set at p<0.05.

### Indirect immunofluorescent staining and live cell imaging

Vero-E6, C6/36, U4.4 or Ae cells were grown on glass coverslips (13 mm diameter), infected with recombinant viruses and fixed at the time points indicated with 4% paraformaldehyde in PBS. After permeabilization with 0.1% Triton X-100 in PBS the cells were reacted with specific primary antibodies, followed by secondary antibody conjugates. Localisation of the fluorescently labelled proteins was examined at various times post infection using a Zeiss LSM-510 confocal microscope. The widths of 50 rMP12 or rMP12:S-Swap NSs filaments were measured using the LSM Image Browser Software (Carl Zeiss MicroImaging GmbH) and the results presented as the mean and standard error of the mean of the two groups.

Images of infected cell monolayers were visualised for eGFP fluorescence using an EVOS FL Cell Imaging System (AMG, Invitrogen).

## Supporting Information

Figure S1 **Sensitivity of anti-N and anti-NSs antibodies.** BSR-T7/5 cells were transfected with either 0.5 or 1.0 µg of expression constructs pTM1-N or pTM1-NSs expressing the MP12 N or NSs proteins respectively. 48 h post transfection, cell lysates were prepared by the addition of 300 µl lysis buffer (100 mM Tris-HCl, pH 6.8; 4% SDS; 20% glycerol; 200 mM DTT, 0.2% bromophenol blue and 25 U/ml Benzonase (Novagen)) and proteins separated on a 4–12% SDS-PAGE gel (Invitrogen). Proteins were transferred to a Hybond-C Extra membrane (Amersham), and the membrane was blocked by incubating in saturation buffer (PBS containing 5% dry milk and 0.1% Tween 20) for 1 h. The membrane was reacted with anti-N and anti-NSs polyclonal antibodies at concentrations of 1∶1000, 5000, 10000 or 20000 for 1 h at room temperature. This was followed by incubation with horseradish peroxidase (HRP)-labelled anti-rabbit (Cell Signalling Technology). Visualization of detected proteins was achieved using SuperSignal WestPico chemiluminescent substrate (Pierce), followed by exposure to x-ray film.
(DOCX)

Figure S2 **Standard curves for qRT-PCR.** Standard Curves for the S segment genome/antigenome and M segment genome/antigenome. 10-fold serial dilutions from in-vitro transcription generated RNAs (of known concentrations and hence copy number) were used to construct the curves. Calculation shows the gradient and R^2^ value for the curve.
(DOCX)

Figure S3 **Melt curve analysis of PCR products.** Melt curve analysis on the qPCR products for S segment genome (A) and antigenome (B), and M segment genome (C) and antigenome (D). The T_m_ of the S segment genome and antigenome assays were 80.8°C and 82.3°C respectively. The M segment genome and antigenome assays utilized the same primers and produced similar PCR products which ensures that the T_m_'s are identical, 79.3°C
(DOCX)

Table S1 **Oligonucleotides used for RT-PCR.**
(DOCX)

Table S2 **Validation parameters.** Validation parameters of the standard curves. Amplification efficiency was calculated using the following function: E = −1+10^(−1/slope)^
(DOCX)

Table S3 **Ratio of genome to antigenome (shown as a percentage of total) from the qPCR assays for virion extraction RNA.** Data collected for the repeated qPCR assays for BHK-21, C6/36, U4.4, and Ae cells infected with both rMP12 and rMP12:S-Swap viruses. The mean value is for each sample set is shown at the base of the table.
(DOCX)

Table S4 **Ratio of genome to antigenome (shown as a percentage of total) from the qPCR assays for total extraction RNA.** Data collected for the repeated qPCR assays for BHK-21, C6/36, U4.4, and Ae cells infected with both rMP12 and rMP12:S-Swap virus. The mean value is for each sample set is shown at the base of the table.
(DOCX)
