# Supplementary material for: The Consequences of Reconfiguring the Ambisense S Genome Segment of Rift Valley Fever Virus on Viral Replication in Mammalian and Mosquito Cells and for Genome Packaging
Source: PLoS Pathog. 2014 Feb 13;10(2):e1003922. doi: 10.1371/journal.ppat.1003922 (PMC3923772; doi:10.1371/journal.ppat.1003922)
Supplement: Figure S1 — Sensitivity of anti-N and anti-NSs antibodies. BSR-T7/5 cells were transfected with either 0.5 or 1.0 µg of expression constructs pTM1-N or pTM1-NSs expressing the MP12 N or NSs proteins respectively. 48 h post transfection, cell lysates were prepared by the addition of 300 µl lysis buffer (100 mM Tris-HCl, pH 6.8; 4% SDS; 20% glycerol; 200 mM DTT, 0.2% bromophenol blue and 25 U/ml Benzonase (Novagen)) and proteins separated on a 4–12% SDS-PAGE gel (Invitrogen). Proteins were transferred to a Hybond-C Extra membrane (Amersham), and the membrane was blocked by incubating in saturation buffer (PBS containing 5% dry milk and 0.1% Tween 20) for 1 h. The membrane was reacted with anti-N and anti-NSs polyclonal antibodies at concentrations of 1∶1000, 5000, 10000 or 20000 for 1 h at room temperature. This was followed by incubation with horseradish peroxidase (HRP)-labelled anti-rabbit (Cell Signalling Technology). Visualization of detected proteins was achieved using SuperSignal WestPico chemiluminescent substrate (Pierce), followed by exposure to x-ray film. (DOCX) [file ppat.1003922.s001.docx]

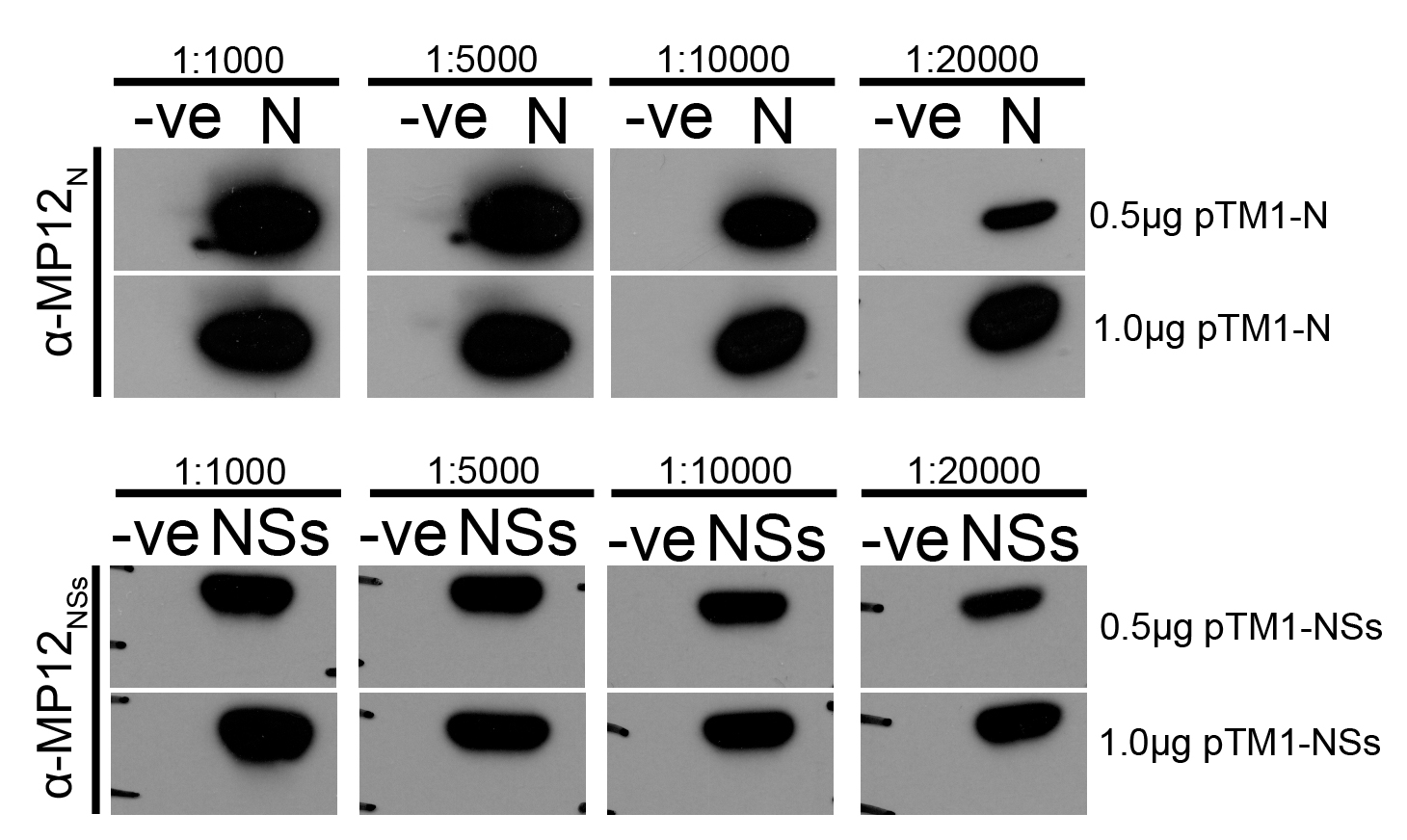


**Figure S1. Sensitivity of anti-N and anti-NSs antibodies.**

BSR-T7/5 cells were transfected with either 0.5 or 1.0 μg of expression constructs pTM1-N or pTM1-NSs expressing the MP12 N or NSs proteins respectively. 48 h post transfection, cell lysates were prepared by the addition of 300 μl lysis buffer (100mM Tris-HCl, pH 6.8; 4% SDS; 20% glycerol; 200mM DTT, 0.2% bromophenol blue and 25U/ml Benzonase (Novagen)) and proteins separated on a 4-12% SDS-PAGE gel (Invitrogen). Proteins were transferred to a Hybond-C Extra membrane (Amersham), and the membrane was blocked by incubating in saturation buffer (PBS containing 5% dry milk and 0.1% Tween 20) for 1 h. The membrane was reacted with anti-N and anti-NSs polyclonal antibodies at concentrations of 1:1000, 5000, 10000 or 20000 for 1 h at room temperature. This was followed by incubation with horseradish peroxidase (HRP)-labelled anti-rabbit (Cell Signalling Technology). Visualization of detected proteins was achieved using SuperSignal WestPico chemiluminescent substrate (Pierce), followed by exposure to x-ray film.
