# Supplementary material for: The Consequences of Reconfiguring the Ambisense S Genome Segment of Rift Valley Fever Virus on Viral Replication in Mammalian and Mosquito Cells and for Genome Packaging
Source: PLoS Pathog. 2014 Feb 13;10(2):e1003922. doi: 10.1371/journal.ppat.1003922 (PMC3923772; doi:10.1371/journal.ppat.1003922)
Supplement: Figure S2 — Standard curves for qRT-PCR. Standard Curves for the S segment genome/antigenome and M segment genome/antigenome. 10-fold serial dilutions from in-vitro transcription generated RNAs (of known concentrations and hence copy number) were used to construct the curves. Calculation shows the gradient and R2 value for the curve. (DOCX) [file ppat.1003922.s002.docx]

Figure S2. Standard curves for qRT-PCR.

Standard Curves for the S segment genome / antigenome and M segment genome / antigenome. 10-fold serial dilutions from in-vitro transcription generated RNAs (of known concentrations and hence copy number) were used to construct the curves. Calculation shows the gradient and R^2^ value for the curve.
