# Supplementary material for: The Consequences of Reconfiguring the Ambisense S Genome Segment of Rift Valley Fever Virus on Viral Replication in Mammalian and Mosquito Cells and for Genome Packaging
Source: PLoS Pathog. 2014 Feb 13;10(2):e1003922. doi: 10.1371/journal.ppat.1003922 (PMC3923772; doi:10.1371/journal.ppat.1003922)
Supplement: Figure S3 — Melt curve analysis of PCR products. Melt curve analysis on the qPCR products for S segment genome (A) and antigenome (B), and M segment genome (C) and antigenome (D). The Tm of the S segment genome and antigenome assays were 80.8°C and 82.3°C respectively. The M segment genome and antigenome assays utilized the same primers and produced similar PCR products which ensures that the Tm's are identical, 79.3°C (DOCX) [file ppat.1003922.s003.docx]

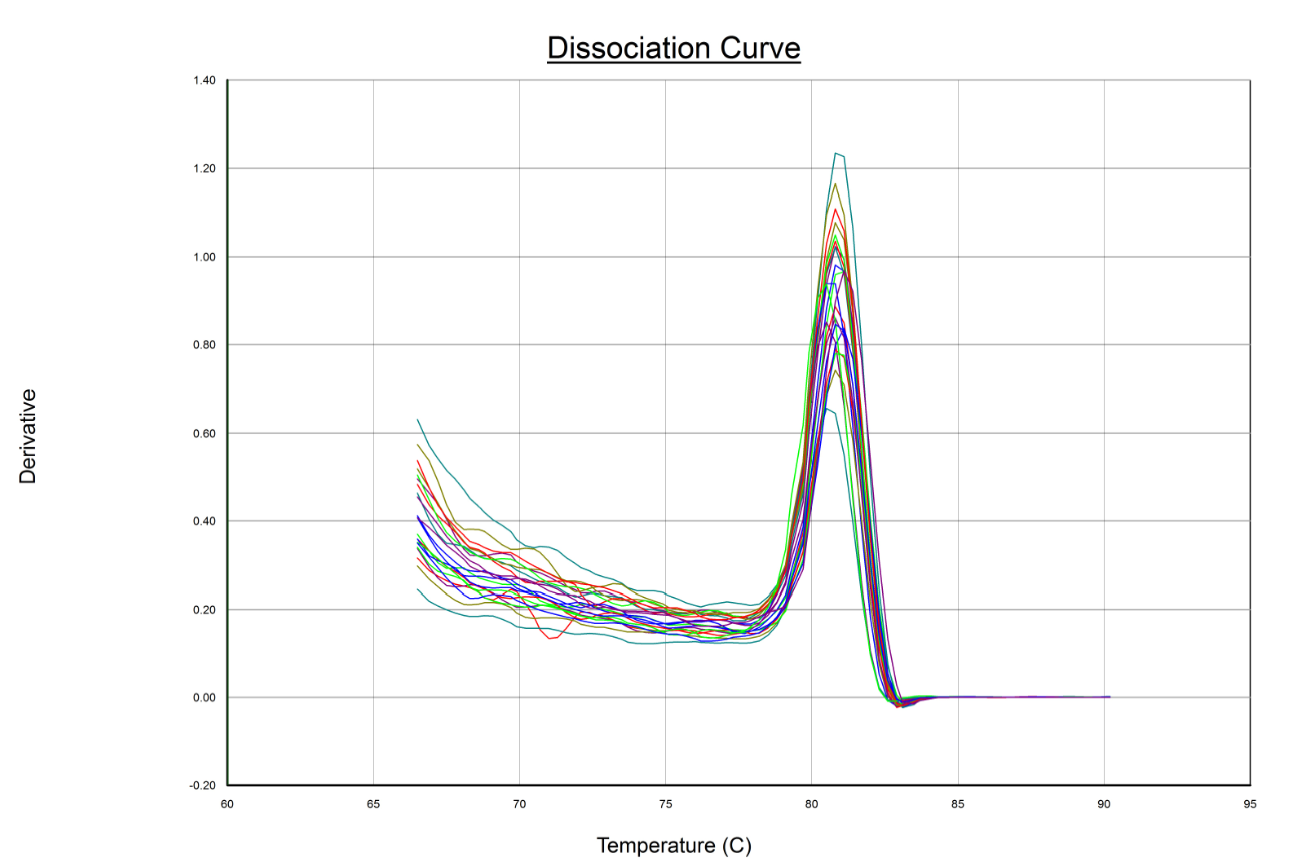

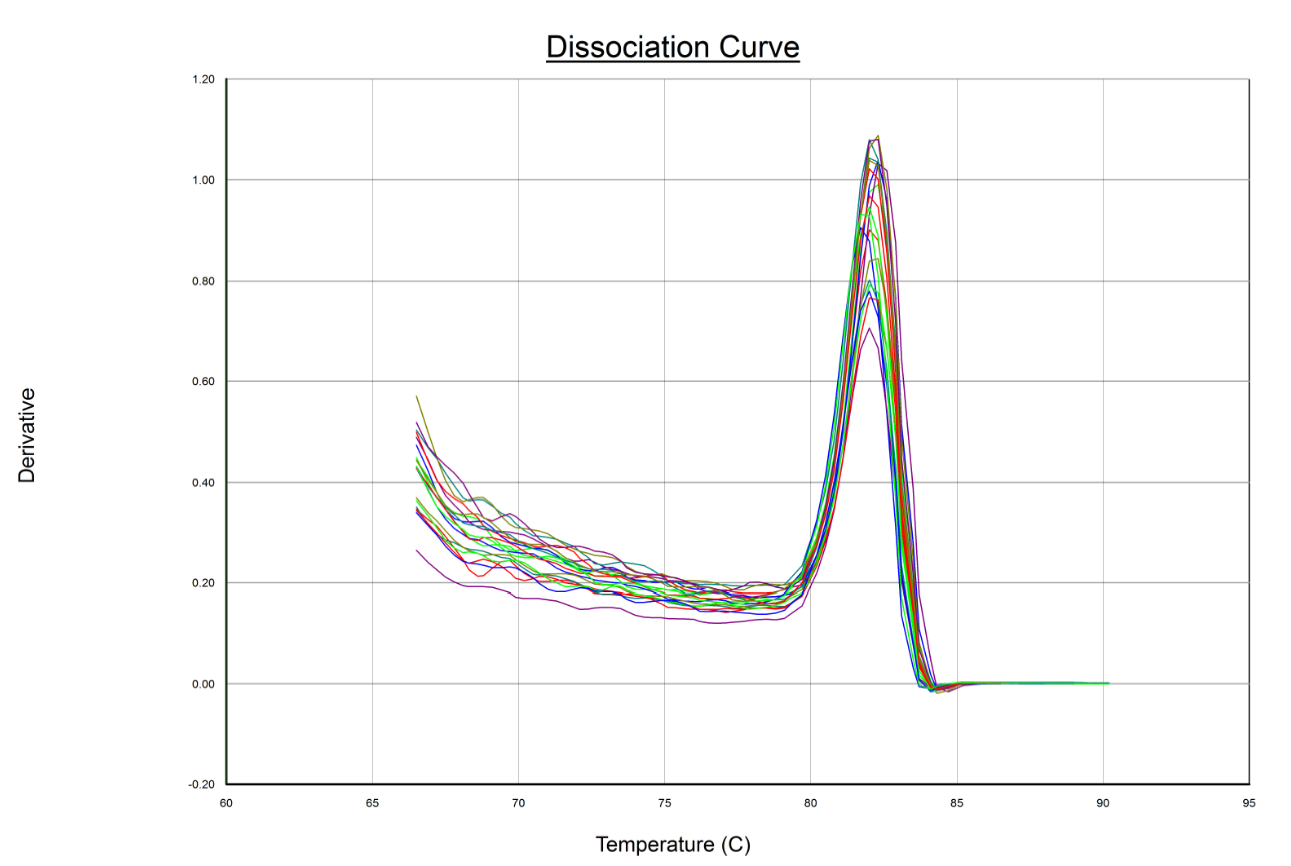

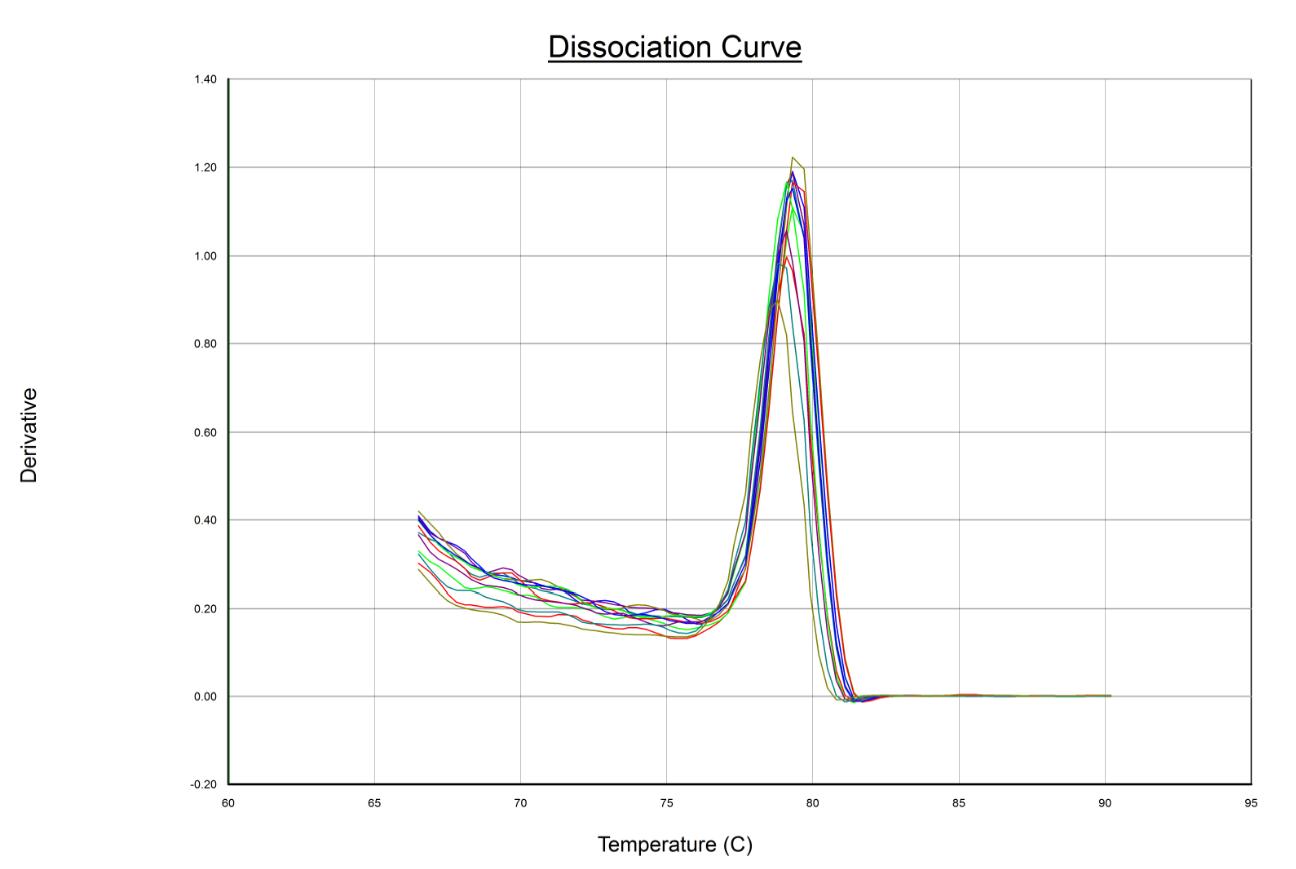

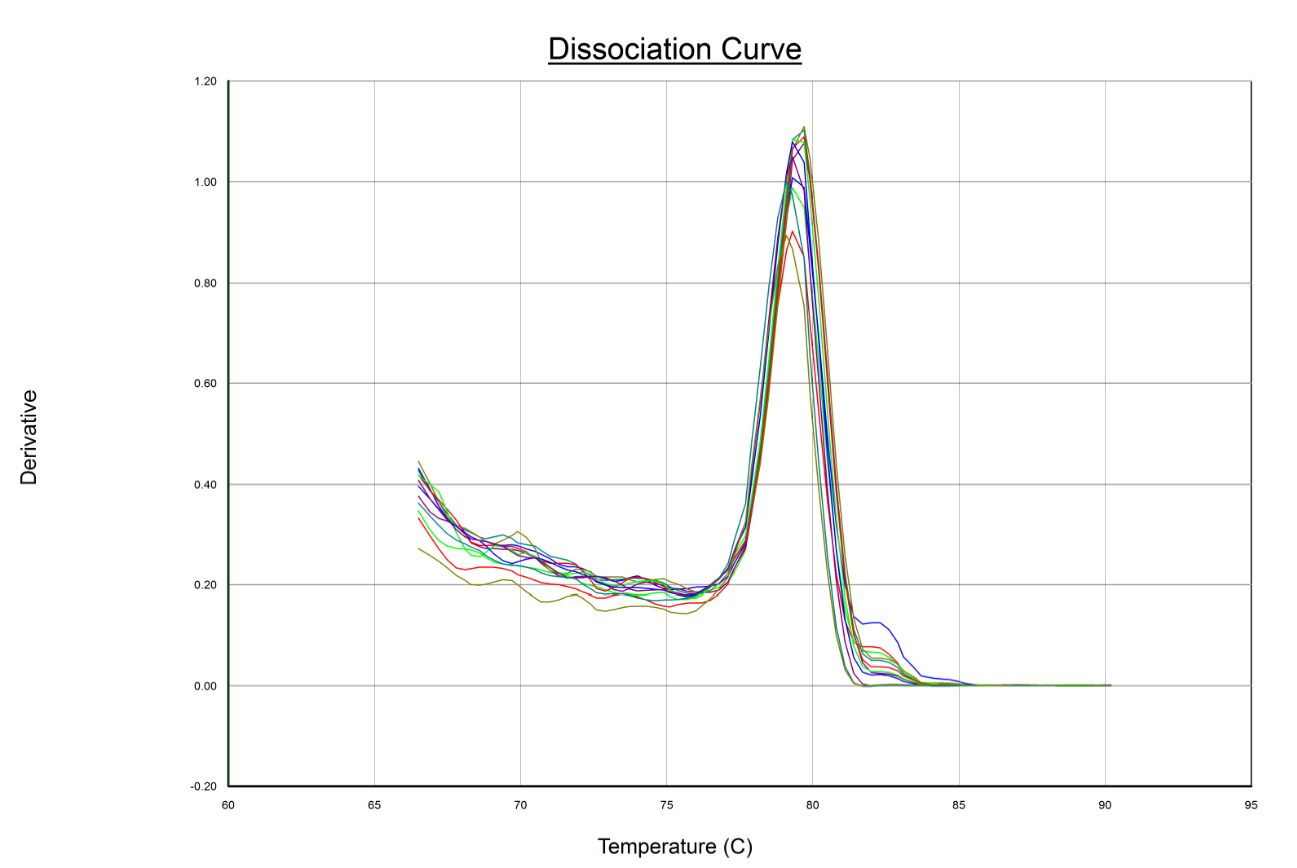


A)

C)

B)

D)

Figure S3. Melt curve analysis of PCR products

Melt curve analysis on the qPCR products for S segment genome (A) and antigenome (B), and M segment genome (C) and antigenome (D). The T_m_ of the S segment genome and antigenome assays were 80.8^o^C and 82.3 ^o^C respectively. The M segment genome and antigenome assays utilized the same primers and produced similar PCR products which ensures that the T_m_’s are identical, 79.3 ^o^C
