# Supplementary material for: The Consequences of Reconfiguring the Ambisense S Genome Segment of Rift Valley Fever Virus on Viral Replication in Mammalian and Mosquito Cells and for Genome Packaging
Source: PLoS Pathog. 2014 Feb 13;10(2):e1003922. doi: 10.1371/journal.ppat.1003922 (PMC3923772; doi:10.1371/journal.ppat.1003922)
Supplement: Table S1 — Oligonucleotides used for RT-PCR. (DOCX) [file ppat.1003922.s004.docx]

| Oligo | Sequence 5’-3’ | Genome Position  (Genomic Sense) |
| --- | --- | --- |
| 1 | ACACAAAGACCCCCTAGTGCTTATC | 1-25 |
| 2 | CTAAGTGGCTGCCCAGGGGGTTG | 896-918 |

**Table S1.** Oligonucleotides used for RT-PCR
