# Supplementary material for: The Consequences of Reconfiguring the Ambisense S Genome Segment of Rift Valley Fever Virus on Viral Replication in Mammalian and Mosquito Cells and for Genome Packaging
Source: PLoS Pathog. 2014 Feb 13;10(2):e1003922. doi: 10.1371/journal.ppat.1003922 (PMC3923772; doi:10.1371/journal.ppat.1003922)
Supplement: Table S2 — Validation parameters. Validation parameters of the standard curves. Amplification efficiency was calculated using the following function: E = −1+10(−1/slope) (DOCX) [file ppat.1003922.s005.docx]

| Segment | Polarity | Slope | Amplification Efficiency (%) | R^2^ |
| --- | --- | --- | --- | --- |
| S | Genome | -3.245 | 103 | 0.996 |
|  | Antigenome | -3.238 | 104 | 0.997 |
|  |  |  |  |  |
| M | Genome | -3.448 | 94 | 0.997 |
|  | Antigenome | -3.538 | 93 | 0.995 |

Table S2: Validation parameters

Validation parameters of the standard curves. Amplification efficiency was calculated using the following function: E = -1+10^(-1/slope)^
