# Supplementary material for: The Consequences of Reconfiguring the Ambisense S Genome Segment of Rift Valley Fever Virus on Viral Replication in Mammalian and Mosquito Cells and for Genome Packaging
Source: PLoS Pathog. 2014 Feb 13;10(2):e1003922. doi: 10.1371/journal.ppat.1003922 (PMC3923772; doi:10.1371/journal.ppat.1003922)
Supplement: Table S3 — Ratio of genome to antigenome (shown as a percentage of total) from the qPCR assays for virion extraction RNA. Data collected for the repeated qPCR assays for BHK-21, C6/36, U4.4, and Ae cells infected with both rMP12 and rMP12:S-Swap viruses. The mean value is for each sample set is shown at the base of the table. (DOCX) [file ppat.1003922.s006.docx]

| **BHK-21** | | | | | | | |
| --- | --- | --- | --- | --- | --- | --- | --- |
| **rMP12** | | | | **rMP12:S-Swap** | | | |
| **S Segment** | | **M Segment** | | **S Segment** | | **M Segment** | |
| **Genome** | **Antigenome** | **Genome** | **Antigenome** | **Genome** | **Antigenome** | **Genome** | **Antigenome** |
| 78.68% | 21.32% | 93.83% | 6.17% | 29.16% | 70.84% | 84.34% | 15.66% |
| 80.87% | 19.13% | 94.93% | 5.07% | 49.80% | 50.20% | 87.78% | 12.22% |
| 83.23% | 16.77% | 94.18% | 5.82% | 42.39% | 57.61% | 91.76% | 8.24% |
| 88.25% | 11.75% | 94.36% | 5.64% | 38.00% | 62.00% | 90.34% | 9.66% |
| 78.77% | 21.23% | 98.13% | 1.87% | 48.99% | 51.01% | 89.21% | 10.79% |
|  |  |  |  | 32.43% | 67.57% |  |  |
|  |  |  |  | 26.21% | 73.79% |  |  |
|  |  |  |  |  |  |  |  |
| **81.96%** | **18.04%** | **95.09%** | **4.91%** | **38.14%** | **61.86%** | **88.69%** | **11.31%** |

| **C6/36** | | | | | | | |
| --- | --- | --- | --- | --- | --- | --- | --- |
| **rMP12** | | | | **rMP12:S-Swap** | | | |
| **S Segment** | | **M Segment** | | **S Segment** | | **M Segment** | |
| **Genome** | **Antigenome** | **Genome** | **Antigenome** | **Genome** | **Antigenome** | **Genome** | **Antigenome** |
| 79.63% | 20.37% | 98.13% | 1.87% | 25.24% | 74.76% | 89.21% | 10.79% |
| 90.32% | 9.68% | 96.93% | 3.07% | 38.70% | 61.30% | 84.16% | 15.84% |
| 83.57% | 16.43% | 98.34% | 1.66% | 4.98% | 95.02% | 88.23% | 11.77% |
| 78.75% | 21.25% | 98.80% | 1.20% | 34.76% | 65.24% | 89.47% | 10.53% |
| 63.21% | 36.79% | 90.70% | 9.30% | 18.43% | 81.57% | 94.37% | 5.63% |
| 85.51% | 14.49% |  |  | 38.12% | 61.88% | 97.17% | 2.83% |
|  |  |  |  |  |  |  |  |
| **80.17%** | **19.83%** | **96.58%** | **3.42%** | **26.71%** | **73.29%** | **90.44%** | **9.57%** |
|  |  |  |  |  |  |  |  |
| **U4.4** | | | | | | | |
| **rMP12** | | | | **rMP12:S-Swap** | | | |
| **S Segment** | | **M Segment** | | **S Segment** | | **M Segment** | |
| **Genome** | **Antigenome** | **Genome** | **Antigenome** | **Genome** | **Antigenome** | **Genome** | **Antigenome** |
| 76.97% | 23.03% | 98.43% | 1.57% | 28.07% | 71.93% | 97.74% | 2.26% |
| 71.15% | 28.85% | 97.39% | 2.61% | 17.83% | 82.17% | 85.30% | 14.70% |
| 80.60% | 19.40% | 98.10% | 1.90% | 33.14% | 66.86% | 96.30% | 3.70% |
| 81.31% | 18.69% | 94.71% | 5.29% | 20.78% | 79.22% | 89.73% | 10.27% |
| 63.05% | 36.95% | 96.27% | 3.73% | 22.65% | 77.35% | 91.64% | 8.36% |
|  |  |  |  |  |  |  |  |
|  |  |  |  |  |  |  |  |
|  |  |  |  |  |  |  |  |
| **74.61%** | **25.39%** | **96.98%** | **3.02%** | **24.49%** | **75.51%** | **92.14%** | **7.86%** |
|  |  |  |  |  |  |  |  |
| **Ae** | | | | | | | |
| **rMP12** | | | | **rMP12:S-Swap** | | | |
| **S Segment** | | **M Segment** | | **S Segment** | | **M Segment** | |
| **Genome** | **Antigenome** | **Genome** | **Antigenome** | **Genome** | **Antigenome** | **Genome** | **Antigenome** |
| 92.61% | 7.39% | 96.98% | 3.02% | 27.34% | 72.66% | 97.20% | 2.80% |
| 67.87% | 32.13% | 98.61% | 1.39% | 21.86% | 78.14% | 81.12% | 18.88% |
| 84.29% | 15.71% | 99.33% | 0.67% | 36.72% | 63.28% | 82.69% | 17.31% |
| 79.34% | 20.66% | 94.56% | 5.44% | 35.86% | 64.14% | 83.44% | 16.56% |
| 85.07% | 14.93% | 94.80% | 5.20% | 28.78% | 71.22% | 92.04% | 7.96% |
|  |  |  |  |  |  | 85.46% | 14.54% |
|  |  |  |  |  |  |  |  |
|  |  |  |  |  |  |  |  |
| **81.84%** | **18.16%** | **96.86%** | **3.14%** | **30.11%** | **69.89%** | **86.99%** | **13.01%** |

**Table S3: Ratio of genome to antigenome (shown as a percentage of total) from the qPCR assays for virion extraction RNA**

Data collected for the repeated qPCR assays for BHK-21, C6/36, U4.4, and Ae cells infected with both rMP12 and rMP12:S-Swap viruses. The mean value is for each sample set is shown at the base of the table.
