# Supplementary material for: The Consequences of Reconfiguring the Ambisense S Genome Segment of Rift Valley Fever Virus on Viral Replication in Mammalian and Mosquito Cells and for Genome Packaging
Source: PLoS Pathog. 2014 Feb 13;10(2):e1003922. doi: 10.1371/journal.ppat.1003922 (PMC3923772; doi:10.1371/journal.ppat.1003922)
Supplement: Table S4 — Ratio of genome to antigenome (shown as a percentage of total) from the qPCR assays for total extraction RNA. Data collected for the repeated qPCR assays for BHK-21, C6/36, U4.4, and Ae cells infected with both rMP12 and rMP12:S-Swap virus. The mean value is for each sample set is shown at the base of the table. (DOCX) [file ppat.1003922.s007.docx]

| **BHK-21** | | | | | | | |
| --- | --- | --- | --- | --- | --- | --- | --- |
| **rMP12** | | | | **rMP12:S-Swap** | | | |
| **S Segment** | | **M Segment** | | **S Segment** | | **M Segment** | |
| **Genome** | **Antigenome** | **Genome** | **Antigenome** | **Genome** | **Antigenome** | **Genome** | **Antigenome** |
| 64.53% | 35.47% | 77.74% | 22.26% | 27.72% | 72.28% | 68.92% | 31.08% |
| 73.73% | 26.27% | 94.84% | 5.16% | 26.24% | 73.76% | 86.10% | 13.90% |
| 61.72% | 38.28% | 93.92% | 6.08% | 18.53% | 81.47% | 90.34% | 9.66% |
| 82.12% | 17.88% | 85.54% | 14.46% | 26.92% | 73.08% | 67.89% | 32.11% |
| 69.98% | 30.02% | 82.33% | 17.67% | 19.93% | 80.07% | 71.33% | 28.67% |
| 79.96% | 20.04% | 84.12% | 15.88% | 21.38% | 78.62% | 72.03% | 27.97% |
|  |  |  |  | 21.53% | 78.47% |  |  |
|  |  |  |  | 32.50% | 67.50% |  |  |
|  |  |  |  |  |  |  |  |
| **72.01%** | **27.99%** | **86.42%** | **13.59%** | **24.34%** | **75.66%** | **76.10%** | **23.90%** |
|  |  |  |  |  |  |  |  |
| **C6/3** | | | | | | | |
| **rMP12** | | | | **rMP12:S-Swap** | | | |
| **S Segment** | | **M Segment** | | **S Segment** | | **M Segment** | |
| **Genome** | **Antigenome** | **Genome** | **Antigenome** | **Genome** | **Antigenome** | **Genome** | **Antigenome** |
| 75.51% | 24.49% | 87.16% | 12.84% | 36.11% | 63.89% | 78.13% | 21.87% |
| 80.85% | 19.15% | 90.04% | 9.96% | 32.58% | 67.42% | 76.26% | 23.74% |
| 99.71% | 0.29% | 81.78% | 18.22% | 36.10% | 63.90% | 76.05% | 23.95% |
| 83.52% | 16.48% | 84.02% | 15.98% | 46.61% | 53.39% | 79.25% | 20.75% |
| 89.35% | 10.65% | 77.81% | 22.19% | 30.09% | 69.91% | 86.74% | 13.26% |
| 68.38% | 31.62% | 93.60% | 6.40% | 36.38% | 63.62% |  |  |
| 96.03% | 3.97% |  |  | 49.93% | 50.07% |  |  |
| 80.88% | 19.12% |  |  |  |  |  |  |
|  |  |  |  |  |  |  |  |
| **84.28%** | **15.72%** | **85.74%** | **14.27%** | **38.26%** | **61.74%** | **79.29%** | **20.71%** |
|  |  |  |  |  |  |  |  |
| **U4.4** | | | | | | | |
| **rMP12** | | | | **rMP12:S-Swap** | | | |
| **S Segment** | | **M Segment** | | **S Segment** | | **M Segment** | |
| **Genome** | **Antigenome** | **Genome** | **Antigenome** | **Genome** | **Antigenome** | **Genome** | **Antigenome** |
| 86.24% | 13.76% | 77.77% | 22.23% | 36.15% | 63.85% | 78.16% | 21.84% |
| 76.60% | 23.40% | 71.96% | 28.04% | 36.29% | 63.71% | 71.02% | 28.98% |
| 73.15% | 26.85% | 69.96% | 30.04% | 41.82% | 58.18% | 71.31% | 28.69% |
| 85.34% | 14.66% | 77.20% | 22.80% | 44.24% | 55.76% | 76.42% | 23.58% |
| 87.53% | 12.47% | 74.10% | 25.90% | 39.80% | 60.20% | 74.10% | 25.90% |
| 79.95% | 20.05% | 71.61% | 28.39% | 32.50% | 67.50% |  |  |
|  |  | 63.11% | 36.89% |  |  |  |  |
|  |  |  |  |  |  |  |  |
| **81.47%** | **18.53%** | **72.24%** | **27.76%** | **38.47%** | **61.53%** | **74.20%** | **25.80%** |
|  |  |  |  |  |  |  |  |
| **Ae** | | | | | | | |
| **rMP12** | | | | **rMP12:S-Swap** | | | |
| **S Segment** | | **M Segment** | | **S Segment** | | **M Segment** | |
| **Genome** | **Antigenome** | **Genome** | **Antigenome** | **Genome** | **Antigenome** | **Genome** | **Antigenome** |
| 79.24% | 20.76% | 79.81% | 20.19% | 17.61% | 82.39% | 84.87% | 15.13% |
| 75.23% | 24.77% | 77.12% | 22.88% | 19.85% | 80.15% | 79.55% | 20.45% |
| 80.07% | 19.93% | 79.25% | 20.75% | 41.15% | 58.85% | 72.10% | 27.90% |
| 77.64% | 22.36% | 82.35% | 17.65% | 12.51% | 87.49% | 79.40% | 20.60% |
| 87.54% | 12.46% | 71.96% | 28.04% | 37.50% | 62.50% | 69.23% | 30.77% |
| 86.40% | 13.60% | 74.41% | 25.59% | 26.98% | 73.02% |  |  |
|  |  |  |  |  |  |  |  |
| **81.02%** | **18.98%** | **77.48%** | **22.52%** | **25.93%** | **74.07%** | **77.03%** | **22.97%** |

**Table S4: Ratio of genome to antigenome (shown as a percentage of total) from the qPCR assays for total extraction RNA**

Data collected for the repeated qPCR assays for BHK-21, C6/36, U4.4, and Ae cells infected with both rMP12 and rMP12:S-Swap virus. The mean value is for each sample set is shown at the base of the table.
